# Supplementary material for: Fast synthesis of large-area bilayer graphene film on Cu
Source: Nat Commun. 2023 Jun 2;14:3199. doi: 10.1038/s41467-023-38877-9 (PMC10238369; doi:10.1038/s41467-023-38877-9)
Supplement: Supplementary file 1 — Supplementary Information [file 41467_2023_38877_MOESM1_ESM.pdf]

Supporting information

Fast synthesis of large-area bilayer graphene film on Cu

Jincan Zhang,[1,2,3,4,†] Xiaoting Liu,[1,2,3,†] Mengqi Zhang,[2,5,†] Rui Zhang,[6,†] Huy Q. Ta,[7] Jianbo Sun,[2] Wendong Wang,[6] Wenqing Zhu,[8] Tiantian Fang,[9] Kaicheng Jia,[1,2] Xiucai Sun,[1,2] Xintong Zhang,[2] Yeshu Zhu,[1,2,3] Jiaxin Shao,[1,2,3] Yuchen Liu,[2] Xin Gao,[1,2,3] Qian Yang,[1,2] Luzhao Sun,[1,2,3] Qin Li,[2] Fushun Liang,[1,2,3] Heng Chen,[1,2] Liming Zheng,[1,2] Fuyi Wang,[10] Wanjian Yin,[11] Xiaoding Wei,[8] Jianbo Yin,[2] Thomas Gemming,[7] Mark. H Rummeli,[7,11,12,13] Haihui Liu,[5*] Hailin Peng,[1,2*] Li Lin,[14*] Zhongfan Liu[1,2*]

[1]Center for Nanochemistry, Beijing Science and Engineering Center for Nanocarbons, Beijing National Laboratory for Molecular Science, College of Chemistry and Molecular Engineering, Peking University, Beijing 100871, P. R. China.
[2]Beijing Graphene Institute, Beijing 100095, P. R. China.
[3]Academy for Advanced Interdisciplinary Studies, Peking University, Beijing 100871, P. R. China
[4]Department of Engineering, University of Cambridge, Cambridge CB3 0FA, UK.
[5]School of Materials Science and Engineering, Tianjin Key Laboratory of Advanced Fibers and Energy Storage, State Key Laboratory of Separation Membranes and Membrane Processes, Tiangong University, Tianjin 300387, P. R. China.
[6]Department of Physics and Astronomy, University of Manchester, Manchester M13 9PL, UK.
[7]Leibniz Institute for Solid State and Materials Research Dresden, P.O. Box 270116, D-01171 Dresden, Germany.
[8]State Key Laboratory for Turbulence and Complex System, Department of Mechanics and Engineering Science, College of Engineering, Peking University, Beijing 100871, P. R. China.
[9]CAS Key Laboratory of Analytical Chemistry for Living Biosystems, Institute of Chemistry, Chinese Academy of Sciences, Beijing, 100190, P. R. China.
[10]Beijing National Laboratory for Molecular Sciences, National Centre for Mass Spectrometry in Beijing, CAS Key Laboratory of Analytical Chemistry for Living Biosystems, Institute of Chemistry, Chinese Academy of Sciences, Beijing, 100190, P. R. China.
[11]Soochow Institute for Energy and Materials Innovations, Soochow University, Suzhou 215006, P. R. China.
[12]Centre of Polymer and Carbon Materials, Polish Academy of Sciences, M. Curie-Skłodowskiej 34, Zabrze 41-819, Poland.
[13]Institute of Environmental Technology, VŠB -Technical University of Ostrava, 17 Listopadu 15, Ostrava, 708 33, Czech Republic.
[14]School of Materials Science and Engineering, Peking University, Beijing 100871, P. R. China.

[#]These authors contributed equally: Jincan Zhang, Xiaoting Liu, Mengqi Zhang, Rui Zhang

*These authors jointly supervised this work: Zhongfan Liu (zfliu@pku.edu.cn), Li Lin (linli-cnc@pku.edu.cn), Hailin Peng (hlpeng@pku.edu.cn) and Haihui Liu (liuhaihui@tiangong.edu.cn)

This supplementary information includes:

Supplementary note 1-12

Supplementary Fig. 1-36

Supplementary Table 1

Supplementary References (1-59)

Supplementary Note 1. Fast synthesis of bilayer graphene (BLG) assisted by CO_2.

As shown in Supplementary Fig. 1a, the BLG film was grown on 25 μm thick Cu foils using a low-pressure hot-wall chemical vapor deposition (CVD) system that was equipped with a quartz tube (6 inch in diameter). For efficient surface cleaning of the commercial Cu foils, CO_2 gas with large flux (500 sccm) was introduced during the heating and annealing stages. Small amounts of CO_2 (no larger than 30 sccm) were then utilized by mixing with H_2 and CH_4 for the high-temperature synthesis of BLG. Note that CO_2 was turned off to avoid graphene etching during the cooling stage (Supplementary Fig. 1b).

The as-synthesized graphene samples were then transferred onto SiO_2/Si substrates for quantitative estimation of the bilayer coverage of graphene grown using different CVD parameters (Supplementary Figs. 2-5). The growth pressure has significant impact on the layer number of graphene films grown on Cu. Specifically, the graphene film was dominated by monolayer and bilayer when the growth pressure was set below 2,000 Pa (Supplementary Fig. 2a,b), while a large amount of few-layer graphene domains appeared when the growth pressure was over 10,000 Pa (Supplementary Fig. 2c). Moreover, when graphene growth was conducted at 1 atm, only isolated thick graphene domains were obtained, as reflected by the varied contrast (Supplementary Fig. 2d). Therefore, growth pressure no higher than 2,000 Pa was utilized to obtain continuous BLG with high uniformity and high growth rate in this work.

Impact of H_2 flux on the growth behaviors of BLG on Cu surface was also investigated. As shown in Supplementary Figs. 3a and 4, the BLG coverage was no higher than 50% when the H_2 flux was set below 300 sccm or above 1,000 sccm. 500 sccm H_2 was thus selected for the BLG growth in this work.

The bilayer coverage increased monotonously with the flux of CH_4 (Supplementary Figs. 3b and 5). However, excess carbon source supply would result in the formation of thicker graphene layers. For example, about ~ 30% few-layer graphene coverage was observed when

using 20 sccm CH_4. Note that when CO_2 or the mixing gases of CO_2 and H_2 flow into the CVD system, no graphene film can be synthesized, indicating that CO_2 cannot function as the carbon source to grow graphene in this work.

Dependence of the bilayer coverage, nucleation density, and domain size on the flux of CO_2 was also studied and the statistical results are shown in Supplementary Fig. 3c,d and Fig. 1d. The BLG nucleation density, which is influenced by both the amount of active nucleation sites on Cu substrate and the amount of active carbon species diffusing between the first-layer graphene and Cu, firstly reached a maximum value at 15 sccm CO_2 and then deceased to a nearly constant value (Supplementary Fig. 3c), while the average domain size of the second-layer graphene increased monotonously with the increased CO_2 flux (Supplementary Fig. 3d). In all, a nearly linear relationship between the bilayer coverage of graphene and the CO_2 flux was achieved (Fig. 1d). In addition, the introduction time of CO_2 also plays a similar role in controlling the bilayer coverage (Fig. 1e,f). Especially, when 500 sccm H_2, 5 sccm CH_4 and 30 sccm CH_4 was utilized, continuous BLG with average domain size ~ 30-50 μm can be obtained in 20 min.

Supplementary Note 2. The role of CO_2 in fast synthesis of BLG.

Compared with H_2, which has also been reported to be able to etch graphene, CO_2 has irreplaceable advantages in the fast synthesis of BLG. This is mainly because CO_2 has a much stronger graphene etching ability[1] and can thus create abundant defect sites in the top graphene layer for the fast diffusion of carbon species to grow the second graphene layer. It has been reported that H_2 tends to etch graphene from its defective regions, such as the edges[2] or the nucleation sites[3,4], while CO_2 attacks both the graphene edge and surface[1,5]. A recent work also reported that H_2 prefers to etch the second graphene layer rather than the top graphene layer because of the existence of abundant active carbon species atop the first layer and the relatively

weaker etching ability of H_2[6].

We have conducted first-principle simulations to compare the graphene etching ability of CO_2 and H_2 from two aspects, the strength of their interactions with graphene (Supplementary Fig. 6) and the thermodynamic changes in energy during graphene etching reactions (Supplementary Fig. 7). First, from the perspective of molecular interactions, the CO_2 on graphene shows a much lower adsorption energy (E_{ad} = 0.07 eV) than that of H_2 (E_{ad} = 0.76 eV) (Supplementary Fig. 6a,c), indicating that the synthetic graphene surface could preferentially capture CO_2 molecules rather than H_2. Further charge density difference (Supplementary Fig. 6b,d) and the Crystal Orbital Hamilton Populations (COHP) calculations (Supplementary Fig. 4e-h) demonstrate a much stronger chemical interaction between CO_2 and graphene, due to the more charge transfer and lower integrated COHP (-ICOHP < -6.0 eV) values between adsorbed CO_2 and graphene atoms. Therefore, as compared with H_2, the CO_2 molecules are more likely to be captured by graphene surface and thus more likely to induce structural deformation and etching reactions driven by electron redistribution. Secondly, from the thermodynamic aspect of etching reaction, the formation energy (E_f) of CO_2 etching graphene is much lower than that of H_2 (Supplementary Fig. 7), which means that even though H_2 can etch the top graphene layer, it is less energy-favorable than CO_2 and thus is more difficult to happen.

Moreover, the introduction of trace CO_2 also contributes to the rapid formation of abundant active carbon species by releasing O atoms[1,7] to decrease the energy barriers of carbon source (CH_4) decomposition in the high-temperature CVD system[8]. As a result, large-area BLG film can be obtained (Supplementary Fig. 8). The contrast difference of bare SiO_2/Si substrate, MLG and BLG is clearly seen in Supplementary Fig. 8a, while for intact regions, a uniform contrast in at least mm-sized areas is observed (Supplementary Fig. 8b).

In contrast, if no CO_2 was used during the graphene growth stage, only the growth of monolayer graphene was observed, as revealed by the OM image of the transferred graphene film on SiO_2/Si substrate and the SAED pattern of the suspended graphene transferred onto the commercial transmission electron microscopy (TEM) grid (Supplementary Fig. 9).

Supplementary Note 3. Layer number control during graphene growth on Cu with the aid of CO_2.

To grow trilayer graphene (TLG) or tetra-layer graphene, abundant defective structures in the bottom graphene layer of the pre-formed BLG or TLG are required so that carbon species could diffuse across the defects and enter the interface between the bottom graphene layer and Cu. However, with the increased graphene thickness, it is more difficult for CO_2 to etch bilayer or multilayer graphene, which is supported by both of our experimental results and theoretical calculation results. According to our experimental results, the nucleation density, growth rate and coverage of TLG and tetralayer graphene are much lower than those of BLG (Supplementary Figs. 2-4), which enables the large-area fast synthesis of BLG film on Cu after optimizing CVD parameters.

First principle simulations have also been conducted by constructing the MLG, BLG and TLG structures on Cu(111) surface to quantitatively investigate the difference of CO_2 etching ability on multilayer graphene (Supplementary Fig. 10). We know that the etching rate of graphene (V_g) at a specific temperature depends on the etching difficulty of single carbon atoms in graphene (V_c) and the flux of etching gas (F_{CO_2}), scilicet as: $V_g \propto V_c * F_{CO_2}$. Since the chemical environment and coordination structure of all C atoms in the perfect multilayer graphene are the same, we believe that the etching rate of C atoms in each graphene layer is the same and can be set as constant under specific CVD conditions. Therefore, the etching difficulty of graphene with different layers only depends on the effective F_{CO_2} contacting it. Further on, the

F_{CO_2} can be calculated by the concentration of CO_2 in contact with graphene of different layers and the corresponding effective exposure area (*S*), which can be written as equation (1):

$$F_{CO_2} = S * e^{-\frac{E_{ad}}{k_B * T}} \quad (1)$$

where k_B and T are Boltzmann constant and experimental temperature of 1,300 K, respectively. As illustrated in Supplementary Fig. 10a-d, the adsorption energy (E_{ad}) of CO_2 molecules on the MLG, BLG, and TLG surfaces were calculated to be -0.07 eV, -1.44 eV, and -2.27 eV, respectively, which indicates the reduced concentration for CO_2 to be captured by BLG or TLG in comparison with the MLG. At the same time, the exposure area (*S*) of the bottom graphene layer to CO_2 also decreases with the layer number. Bringing into the numerical calculation, we can obtain the F_{CO_2} values (Supplementary Fig. 10e). It is clearly observed that the etching flux of graphene decreases significantly with its layer number. Moreover, since the real defect density of BLG and TLG is much lower than that estimated from the calculation model we built here (Supplementary Fig. 10b,c), the formation of TLG and tetra-layer graphene with the aid of CO_2 is thus even more difficult.

Supplementary Note 4. Mass production of BLG on commercial Cu foils with the aid of CO_2

Batch-to-batch production of large-area BLG film was conducted using commercial polycrystalline Cu foils without special pre-treatment. To evaluate the bilayer coverage, OM images of the transferred BLG were acquired every 1 cm (Supplementary Fig. 11) using 10X objective under optical microscope, which cover sub-mm sized area for each image. To provide more details about the BLG, OM images were also taken using x50 objective after annealing the samples to decrease disturbance of the transfer-induced polymer residues (Supplementary Fig. 12). Note that the areal ratio of BLG was confirmed based on its contrast difference from that of MLG or few-layer graphene and for all measured positions, >94% coverage of BLG

films was observed, indicating that the self-limited growth of graphene on Cu has been broken after introducing CO_2.

Furthermore, using a home-made roll-to-roll system, 2 m long Cu foil was also utilized for the fast synthesis of BLG (Supplementary Fig. 13). According to Supplementary Fig. 13a,b, large-area graphene film with an average bilayer coverage of ~ 92% was obtained after the introduction of CO_2 while the bilayer coverage of BLG was ~ 30% when no CO_2 was utilized. Meanwhile, similar impacts of the fluxes of H_2, CH_4 and CO_2 on the growth behaviors of graphene on Cu foil were observed (Supplementary Fig. 13b,d-f).

Supplementary Note 5. Growth dynamic of the BLG.

To investigate the growth dynamic of BLG films, isotropic-labelled $^{13}CH_4$ was employed[9,10] and the bilayer coverage was tuned by controlling the high-temperature growth time. As shown in Supplementary Fig. 14a, after sequentially flowing ^{12}C and ^{13}C during the graphene growth stage, BLG, which was composed of ^{12}C in the first layer and consisted of both ^{12}C and ^{13}C in the second layer, was synthesized. Continuous MLG film was quickly formed in the first 2 min, followed by the appearance and rapid enlargement of BLG domains, as indicated by the clearly visible alternating ^{12}C and ^{13}C isotropic rings (Fig. 2e and Supplementary Fig. 14b).

Since the carbon source was changed every 2 min, the nucleation and merging time of the BLG can thus be estimated according to the number of the isotropic rings. As shown in Supplementary Fig. 14c,d, nucleation of BLG occurred mainly in the first 15 min while merging of the adjacent BLG domains happened mainly in the last 5 min. Notably, the growth rate of BLG during the coalescence stage was not slowed down, indicating the sufficient supply of the active carbon species.

Furthermore, growth dynamic of the second-layer graphene crystals was also investigated (Supplementary Fig. 15). The domain size of the BLG was defined as the length of the diagonal

in the approximately hexagonal shaped domain. Through Raman line scanning with the step of 2 μm, Raman spectra along the diagonal direction of the BLG domain were acquired (Supplementary Fig. 15c), verifying the alternative appearance of $^{12}C/^{12}C$-BLG and $^{12}C/^{13}C$-BLG. Moreover, after the Raman map of the half BLG domain, ^{13}C-I_G was shown in Supplementary Fig. 15d. Accordingly, the relationship between the domain size and the growth time of the BLG was then plotted in Supplementary Fig. 15e, verifying a nearly constant growth rate of the BLG (~ 2 μm/min, i.e., 120 μm/h).

Supplementary Note 6. Stacking sequence of the BLG.

Stacking sequence of the BLG was identified by performing mild Bi^{3+} sputtering to acquire the depth profile of ^{12}C and ^{13}C using time-of-flight secondary ion mass spectrometry (ToF-SIMS). Except for the distribution of $^{13}C^{2-}$ and $^{12}C^{2-}$ based on 0-2 s, 11-13 s, and 0-15 s accumulation (Fig. 2g and Supplementary Fig. 16a,b), the ring-like patterns of $^{13}C^{4-}$ and $^{12}C^{4-}$ after 11-13s and 0-15s accumulation also confirmed that the second layer graphene is grown underlying the top layer (Supplementary Fig.16c,d).

To study the stacking sequence of the BLG, mild oxygen plasma etching, which was carefully optimized to etch the top layer graphene without destroying the bottom layer[6,11], was conducted using a ^{13}C isotropic-labelled BLG sample transferred onto a SiO_2/Si substrate, which was synthesized by alternatively introducing $^{13}CH_4$ and $^{12}CH_4$ every 2 min for 10 min (Supplementary Fig. 17a). Raman mapping images of ^{13}C-I_G and ^{12}C-I_G confirmed the continuous distribution of ^{12}C in the whole region and the appearance of ^{13}C in the BLG regions, which appeared in the shape of two hexagonal rings (Supplementary Fig. 17b,c). The stronger intensity of ^{12}C-G band also corresponded to the BLG regions. After plasma treatment, Raman spectra of the ^{12}C-MLG and the $^{12}C/^{13}C$-BLG with different mass ratio of ^{12}C over ^{13}C in the second layer were then extracted, as indicated by the varied ratio of ^{13}C-I_G to ^{12}C-I_G (^{13}C-I_G/^{12}C-

I_G) (Supplementary Fig. 17d). Appearance of ^{12}C-labelled D band in both the MLG and BLG regions and absence of ^{13}C-labelled D band (Supplementary Fig. 17e) implied that the second graphene layer is grown beneath the first graphene layer. The phenomenon that the ^{13}C-I_{2D}/^{12}C-I_{2D} was not directly proportional to the ^{13}C-I_G/^{12}C-I_G provided another indication of the poor crystallinity of the continuous top graphene layer, which was composed of ^{12}C after oxygen plasma treatment, as the 2D band intensity is more sensitive to graphene crystallinity. In addition, the ring-like patterns of ^{12}C and ^{13}C elements in both the ToF-SIMS and Raman measurement results also exclude the contributions of C atoms from CO_2 to graphene growth, even though the flux of CO_2 is much higher than that of CH_4.

Supplementary Note 7. Formation and healing of defects in first-layer graphene.

When CO_2 is introduced into the CVD system during the high-temperature growth stage, it etches graphene[5] Etching of graphene by CO_2 was also clearly observed in this work. Firstly, after the formation of continuous MLG on Cu and then the interrupted supply of CH_4 and H_2, hexagonal-shaped holes would be formed by CO_2 (30 sccm) etching for 1 min (Supplementary Fig. 18a). Secondly, when 1 sccm CH_4 and 500 sccm H_2 was utilized for 20 min, continuous graphene film can be obtained on Cu surface, while the introduction of CO_2 led to the formation of isolated MLG domains (Supplementary Fig. 3b). Thirdly, the formation of point defects in the continuous MLG film was also detected by Raman spectroscopy and the defect density was found to be closely related with the CO_2 flux (Supplementary Fig. 18b). The uniformly distributed defects then provide channels for active carbon species to diffuse between the first-layer graphene and the Cu underneath, which enabled the fast nucleation and growth of the second-layer graphene underneath the first layer graphene[12]. Notably, the defects were further repaired during or after the growth of the second layer graphene, as indicated by the downshift of the ^{12}C-G band in the ^{13}C/^{12}C BLG (Supplementary Fig. 18c). Moreover, the rapid

termination of the BLG growth after turning off CO_2 also indicates the efficient healing of the defective structures in the first-layer graphene (Fig. 1f), in good agreement with previous reported results[7,13,14].

We have conducted first-principle calculations using the model of BLG/Cu to explore the elementary reaction of C active species (dominated by CH particles) from free state to splicing to the upper and lower layers of graphene respectively, and evaluate the self-healing mechanism of BLG process according to the structural evolution and energy profiles (Supplementary Fig. 19). In general, the CH species used for graphene growth can be from the gas phase (Supplementary Fig. 19a) or the catalyst surface (Supplementary Fig. 19b). For both conditions, we found an obvious energy decrease of > 7.4 eV after the CH species reaches a stable adsorption state, indicating that the carbon species can be easily captured by graphene defect edges and thus supplies the essential C atoms to form perfect graphene. Moreover, the splicing energy of CH at the edge of the upper and lower graphene defects is almost equal, which means that the defect healing of the upper graphene is almost synchronous with the growth of the lower graphene. After that, rapid self-healing of the defective graphene at high temperature on Cu substrates will happen[12,15].

To further verify the role of the point defects in first-layer graphene for the fast synthesis of BLG, oxygen plasma treatment was utilized to create small defect sites in the MLG film on Cu with varied density. The defective MLG was then put back into the CVD system for the second growth of graphene under the mixing gas of CH_4 and H_2. To avoid the etching of graphene during the temperature raising stage in the atmosphere of H_2, the samples were not put into the heating region until the center of the quartz tube in the furnace reached the growth temperature (~1,000 ^{o}C). After the second growth, the dependence of the BLG coverage on the plasma treatment time was clearly observed, as revealed by the SEM characterization results (Supplementary Fig. 20). Considering that MLG etched with longer time has higher defect

density, it can thus be concluded that the formation of more defects in the MLG before the growth of the second layer graphene resulted in higher BLG coverage. Note that comparing with the plasma etching method to introduce defect sites in graphene lattice, our CO_2-assited approach is more compatible with the mass production processes, with regards to the good controllability in graphene domain size and layer number, as well as the time-saving synthesis process.

Supplementary Note 8. TEM characterization of the BLG film grown on Cu(100)-dominated polycrystalline Cu foils.

The crystallinity and stacking order of the BLG were investigated after transfer onto 3 mm-sized TEM grids. For each sample, more than 50 selected area electron diffraction (SAED) patterns were acquired on different positions across the whole region of the 3 mm-sized TEM grid across the whole 3 mm-sized TEM grid, which distribute uniformly in large area, rather than concentrating on some special regions. The stacking structure of BLG is found to be closely related with the crystalline plane of the metal substrates. Specifically, when the Cu(100)-dominated polycrystalline Cu substrate was utilized for graphene growth, ~ 61% regions were dominated by AB-stacking structures (Supplementary Fig. 21).

For the same BLG film grown on Cu(100)-dominated polycrystalline Cu foils, after acquiring SAED patterns, HRTEM images from positions far away from each other (adjacent distance > 500 μm) were acquired across the 3 mm-sized TEM grid, from which the point defects were seldom observed, further implying high crystallinity of the BLG. Note that in addition to the three AB-BLG regions, six tBLG regions with different twist angles were also characterized using HRTEM (Supplementary Fig. 22).

Supplementary Note 9. Nano-indentation measurement of the BLG.

To assess the mechanical property of the as-synthesized BLG, the suspended graphene was transferred on the holey SiO_2/Si substrate, on which there are circular holes with diameter of 3-5 μm and depth of 300 nm. The AFM nano-indentation experiment was then conducted to measure the force-displacement curves using a single-crystal diamond probe (ART D300, SCD Probes), which has a radius of ~ 10 nm, and a calibrated cantilever stiffness of 30.85 $N \cdot m^{-1}$, following the Sader method[16]. During the measurement, large-area zoom-in topography scanning was firstly conducted to find the position of the suspended graphene membrane and then the AFM tip was located at the center of suspended region before collecting its force–depth responses through indentation by using a constant displacement rate of 500 $nm \cdot s^{-1}$.

As reported previously[17], the force–depth behavior of graphene can be fitted using the following non-linear model (equation 2, 3):

$$F = \left(\pi\sigma_0^{2D}\right)\delta + \left(E^{2D}\frac{q^3}{r^2}\right)\delta^3 \tag{2}$$

$$\sigma = \frac{1}{t}\sqrt{\frac{F_b E^{2D}}{4\pi r}} \tag{3}$$

where F is the applied load; σ_0^{2D} is the two-dimensional pre-stress, δ is the indentation depth; E^{2D} is the Young's modulus of the measured graphene nanosheet; r is the radius of micro-holes; F_b is the breaking force; t is the thickness of the graphene. In addition, the value of the dimensionless constant q is 1.02 for graphene[17]. Specifically, the force range of 0 - 100 nN was utilized for the non-linear fitting in this work. Taking the force–depth curve in Fig. 3f as an example, since the size of the suspended BLG is 5 μm in diameter, the Young's modulus of the BLG was then calculated to be ~698 N m^{-1}, corresponding to 1.04 TPa when considering the thickness of BLG of 0.67 nm and the tip radius of 10 nm. The fracture strength of BLG was calculated to be 79.5 N m^{-1}, corresponding to 118.6 GPa, much higher than that of the MLG.

Note that the size of the suspended MLG is 3 μm in diameter and the Young's modulus of the MLG was calculated to be 218 N m^{-1} (Supplementary Fig. 23).

Supplementary Note 10. Transmittance and sheet resistance measurement of the polycrystalline BLG in large area.

To conduct the transmittance mapping of the MLG and BLG samples in large area, we firstly transferred them onto quart substrates and then manually moved the samples every 1 cm in both horizontal and vertical directions to acquire their transmittance at 550 nm wavelength using a commercial UV-vis spectrometer. For each sample, 9 positions were measured to evaluate its uniformity in ~3 x 3 cm^2-sized region (Supplementary Fig. 24).

To measure sheet resistance, the BLG film grown on polycrystalline Cu was utilized and comprised ~60% AB-BLG and 40% tBLG by area. The sheet resistance of the graphene was directly measured using CDE RsMap 178 after being transferred onto SiO_2/Si substrates. The distance between the 4 pins is 1 mm.

There are several reasons for the observed reduced sheet resistance of BLG in comparison with MLG. Firstly, according to the equation (4)

$$\sigma = n * e * \mu \tag{4}$$

the electrical conductivity (σ) of graphene is decided by both the carrier mobility (μ) and carrier concentration (n). For BLG, even though the carrier mobility decreased in comparison with MLG, the increased number of conduction channels would contribute to the increased carrier concentration and thus increases the electrical conductivity[18]. The increased electrical conductance of BLG has been widely investigated and was supported by both theoretical simulation and experimental results[19-23]. Secondly, our BLG is high quality and has low defect density, as indicated by the negligible D band in Raman spectra, which also contributes to the improved electrical conductivity[24]. Thirdly, the BLG used for sheet resistance measurement is

grown on the Cu(100)-dominant polycrystalline Cu and is composed of ~ 61% AB-BLG and ~39% tBLG by area; the existence of tBLG areas with high twist angles also contributes to decreasing sheet resistance of the BLG film, as reported by Yuji Araki *et al.*[25]. The enhanced mechanical property of BLG (Fig. 3c) and the optimized transfer process can reduce the possibility of crack formation during transfer compared with that of MLG, which in turn benefits the reliable measurement of sheet resistance values in large area. Moreover, similar to previous reported result[26], *p*-doping effect was observed in our BLG samples after the PMMA-assisted wet transfer (Supplementary Fig. 25)[27], which also contributes to decreasing the sheet resistance of BLG films.

Supplementary Note 11. Synthesis and characterization of the AB-BLG grown on Cu(111) substrate.

To improve the ratio of AB-BLG and reduce the non-AB ratio, two kinds of single-crystal Cu(111) substrates were used, including the cm-sized Cu(111) foils which derived from the high-temperature annealing of the commercially available Cu foil (i.e. Cu(111) foil)[28] and the ultraflat single-crystal Cu(111) substrate that was obtained by epitaxial growth of Cu(111) (i.e. Cu(111) film) on annealed c-plane sapphire[29]. For Cu(111) foils, continuous BLG film can be grown within 20 min and the ratio of AB-BLG is up to 96% and this ratio might be further increased to 100% by further improving the purity[30] and surface smoothness[31] of Cu(111) foils. In this work, to further improve the AB-BLG ratio to 100%, the ultraflat single-crystal Cu(111) substrate on c-plane sapphire was used. The growth of this bilayer graphene films requires 90 min, because the graphene growth on the 500 nm thick Cu film needs to be conducted under atmosphere pressure to minimize Cu sublimation, and CO_2 needs to be introduced after the formation of continuous MLG owing. Otherwise, no continuous graphene can be obtained owing to the increased etching ability of CO_2 which is caused by its decreased flow velocity

and thus the longer staying time of CO_2 in the atmosphere-pressure CVD system. Since the amount of CH_4 used for growing MLG was set small to avoid the formation of multilayer graphene in this stage, a longer time was consumed in the growth of the graphene layer. According to our previous experiences in BLG growth on Cu foils (Supplementary Fig. 3), it is also possible to further decrease the growth time of 100% AB-BLG on Cu(111) film by optimizing the CVD parameters, such as increasing the CH_4 concentration in the Ar gas and fine tuning the H_2, CH_4, and CO_2 ratio.

TEM and SAED characterizations were first performed to evaluate the stacking structures of the BLG film grown on Cu(111) substrates. Note that SAED patterns were acquired across the whole TEM grid to evaluate the mono-crystallinity of the synthesized BLG film and the ratio of AB-stacking BLG (AB-BLG) structure increased from <65% on polycrystalline Cu to ~96% (Supplementary Fig. 26) by using the Cu(111) foils prepared via high-temperature annealing[26]. OM image of isolated BLG domains grown on Cu(111) foil was also acquired after transferring graphene onto SiO_2/Si substrate, considering rough surface of the Cu(111) foils. After that, well aligned isolated BLG domains with near-hexagonal shapes can be clearly observed (Supplementary Fig. 27).

The continuous BLG film grown on the ultraflat single-crystal Cu(111) substrate that was obtained by epitaxial growth of Cu(111) (i.e. Cu(111) film) on annealed c-plane was also transferred to TEM grid for SAED characterizations. 100% AB-BLG was obtained on this substrate (Supplementary Fig. 28). Moreover, low energy electron diffraction (LEED) patterns of the AB-BLG film grown on the Cu(111)/sapphire substrate were also acquired (Supplementary Fig. 29). The single set of hexagonal diffraction spots with no rotational misalignment suggests the epitaxial growth of AB-stacking BLG (AB-BLG) on the single-crystal Cu(111) substrate. The identical orientations of the LEED patterns acquired at three different positions across the whole sample (2 x 2 cm^2) further demonstrate the successful

synthesis of the large-scale AB-BLG single-crystal film in at least cm-sized regions. In addition, the OM image of the isolated BLG domains grown on the Cu(111)/sapphire substrate was captured directly without graphene transfer (Supplementary Fig. 30). Note that the irregular shape of graphene domains might be owing to the atmosphere growth pressure.

Raman measurement of the AB-BLG grown on Cu(111) substrate was also conducted after being transferred to SiO_2/Si substrate. Note that the five Raman spectra displayed in Fig. 4b were firstly acquired in the marked positions in Supplementary Fig. 31a through a line mapping with the step of 50 μm and then normalized using the intensity of G bands. Meanwhile, Raman mapping was also conducted in the marked square-shaped region. The distribution of full width at half maximum of 2D band (FWHM(2D)) and the intensity ratio of 2D to G bands (I_{2D}/I_G) were both plotted, based on which the statistical histogram of FWHM(2D) and I_{2D}/I_G was also plotted (Supplementary Fig. 31b,c). Note that the intensity of 2D band is about half of that of the G band, which is a clear indication of the AB-stacking structure in BLG.

To further evaluate the AB-stacking structures of the BLG grown on the ultraflat single-crystal Cu(111) substrate that was obtained by epitaxial growth of Cu(111) (i.e. Cu(111) film) on annealed c-plane sapphire. prepared on c-plane sapphire, Raman characterization has also been conducted to evaluate the AB-stacking structures after transferring the BLG samples onto SiO_2/Si substrates. Note that the Raman spectra were acquired every 1 mm on three cm-sized BLG samples grown on Cu(111)/Sapphire substrates (Supplementary Fig. 32). For all the spectra, the I_{2D}/I_G value is between 0.5 to 1 and the FWHM(2D) value is between 40 to 60 cm^{-1}, furthering confirming the successful preparation of AB-BLG (100% ratio) over the large area.

Supplementary Note 12. Fabrication and measurements of AB-BLG-based electronic and photoelectronic devices.

For the electrical transport measurement, graphene grown on a Cu (111) foil, which was

composed of isolated AB-BLG domains and continuous MLG film was utilized (Supplementary Fig. 33a). After Raman characterization across the whole domain (Supplementary Fig. 33b), the BLG domain was picked up by a hBN nanoflake to fabricate the hBN/BLG/hBN sandwiched structure (Supplementary Fig. 33c). Note that for all the Raman spectra, the I_{2D}/I_G is between 0.5 to 1 and the FWHM(2D) value is between 40 to 60 cm^{-1}, confirming that this BLG domain is AB-stacking structure[32].

After fabrication of the dual-gate BLG Hall bar device, an AC current I_{ds} with a root mean square amplitude of 1 μA at 23.33 Hz was applied between the source and drain terminals. The four-point longitude voltage drop V_{xx} and transverse voltage drop V_{xy} were then measured with lock-in amplifiers. The charge density tuning in the graphene channel was achieved by applying different back gate voltage (V_{bg}) and top gate voltage (V_{tg}). The longitude resistivity ρ_{xx} can be calculated from equation (5):

$$\rho_{xx} = R_{xx} * W/L \tag{5}$$

where W is the width of the conducting channel, L is the length of the channel between the probed contacts, and R_{xx} is the longitude resistance $R_{xx} = V_{xx}/I_{ds}$, and hence the longitude conductivity σ_{xx} can be obtained via equation (6):

$$\sigma_{xx} = 1/\rho_{xx} \tag{6}$$

Firstly, to eliminate the negative effect of oxygen and water in air on the device performance, the carrier mobility of the AB-BLG at 300 K was measured in the glove box (Ar atmosphere), which was calculated to be 5,900 $cm^2V^{-1}s^{-1}$ and 2,700 $cm^2V^{-1}s^{-1}$ for holes and electrons, respectively (Supplementary Fig. 34). Note that, impacts on the electrical transport measurement of the BLG from both the polymer residues atop the graphene, introduced during the wet transfer, and the bubbles formed during the stacking, cannot be ignored, since they severely impact the interface between hBN and graphene, especially considering that the bubble density and size in the BLG region are much larger than those in the MLG regions

(Supplementary Fig. 33c). The polymer residues also result in the *p*-doping of our BLG device[26], even if it is encapsulated by hBN flakes. To further reduce the impact from the measurement environment and surface/interface contaminations, to measure the tunability of the band gap, the device was tested in vacuum at 290 K after keeping it in high vacuum (~ 10^{-7} torr) for one week. In detail, when sweeping the bottom-gate voltage (V_{bg}) from -15 V to 50 V and sweeping the top-gate voltage (V_{tg}) from 4 V to -15 V, the resistance reached maximum at highest displacement field region (the top-left and bottom-right), confirming the tunability of bandgap with perpendicular dipole electric field (Fig. 4d). The transfer curves of the AB-BLG when sweeping the V_{bg} at different values of the fixed V_{tg} is given in Supplementary Fig. 35, in which the maximum resistance attained at the charge neutrality point increases with increasing V_{tg} in both the positive and negative directions, further confirming the opening of the band gap.

The photocurrent measurement was performed in air at room temperature using a two-terminal field effect transistor device. In detail, a 532 nm solid state laser (MGL-III-532, CNI) was utilized as the excitation light source and the light was modulated using a chopper at a chopping frequency of 177 Hz. The photocurrent signal was first converted to a voltage using a current pre-amplifier and then measured by a lock-in amplifier (synchronized with the chopper). The device was mounted on a motorized stage, which allows scanning of the photoresponsivity of the selected device area. The maximum photoresponsivity for the BLG and MLG was estimated to be ~0.089 AW^{-1} and ~ 0.042 AW^{-1}, indicating that a much larger net photocurrent was generated in the BLG/metal hybrid region.

Raman characterization was also conducted after the device fabrication (Supplementary Fig. 36a). Typical Raman spectra of the MLG and BLG are displayed in Supplementary Fig. 36b, confirming the low defect density and AB-stacking structure of the BLG, which was further revealed by the Raman mapping results of I_G, I_{2D}/I_G, and I_D/I_G in Supplementary Fig. 36c-e.

Supplementary Figures

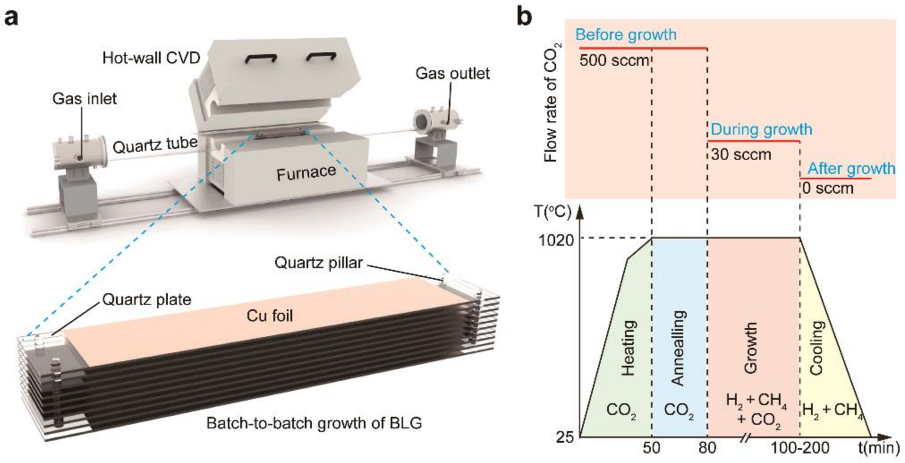

Supplementary Fig. 1. Growth process of the bilayer graphene on Cu with the assistance of CO_2. **a** Schematic diagram of the chemical vapor deposition (CVD) system for the batch production of the BLG films. **b** CVD process for the fast synthesis of BLG film, with CO_2 flux changed at different stages.

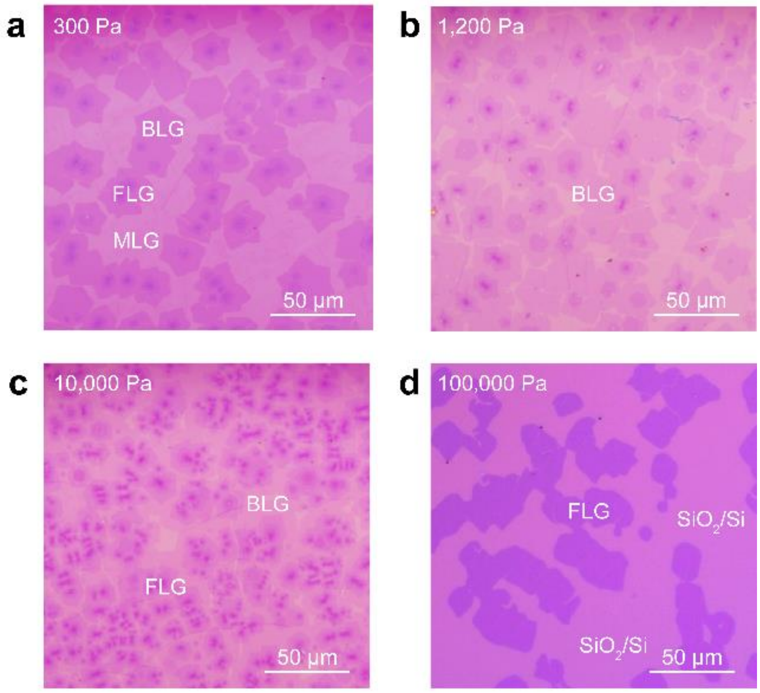

Supplementary Fig. 2. Impact of the growth pressure on the graphene synthesis. a-d Optical microscopy images of the graphene films transferred onto SiO_2/Si substrates, which were prepared under growth pressure of 300 Pa (**a**), 1,200 Pa (**b**), 10,000 Pa (**c**), and 1 atm (**d**). MLG, BLG and FLG

correspond to monolayer graphene, bilayer graphene and few-layer graphene.

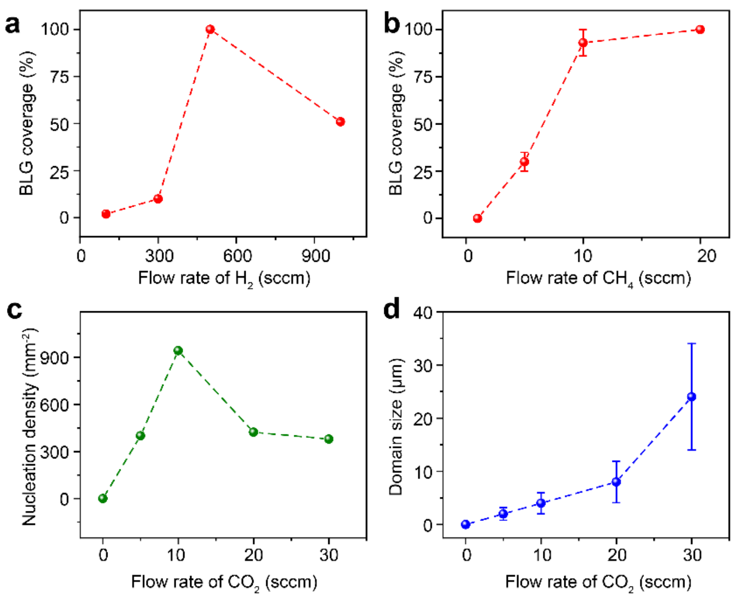

Supplementary Fig. 3. Impact of the gas atmosphere on the growth behavior of the bilayer graphene (BLG). a,b Impact of the H_2 (**a**) and CH_4 (**b**) flow on the BLG coverage. **c,d** Impact of the CO_2 flux on the nucleation density (**c**) and domain size (**d**) of the BLG. Error bars represent standard deviations from three measurement results for each type of sample.

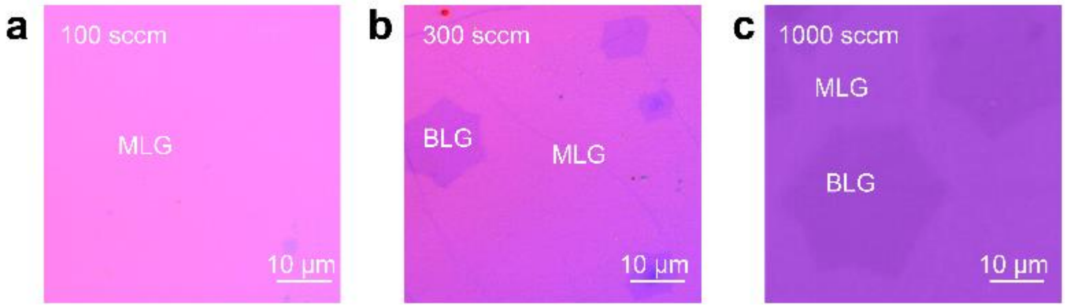

Supplementary Fig. 4. Impact of the H_2 flux on the synthesis of the bilayer graphene (BLG). a-c Optical microscope images of the graphene films transferred onto SiO_2/Si substrates, which were prepared using 100 sccm (**a**), 300 sccm (**b**), and 1000 sccm (**c**) H_2. Note that MLG and BLG are the abbreviations of monolayer graphene and bilayer graphene, respectively.

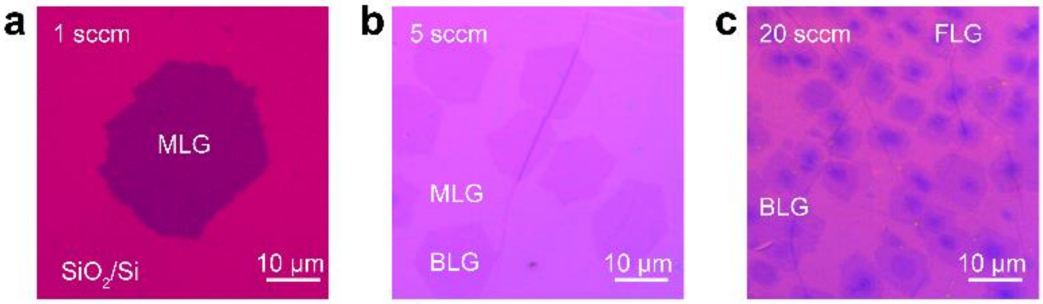

Supplementary Fig. 5. Impact of the CH_4 flux on the synthesis of the bilayer graphene. a-c OM images of the graphene films transferred onto SiO_2/Si substrates, which were prepared using 1 sccm (**a**), 5 sccm (**b**), and 20 sccm (**c**) CH_4. MLG, BLG and FLG in the figure correspond to monolayer graphene, bilayer graphene and few-layer graphene.

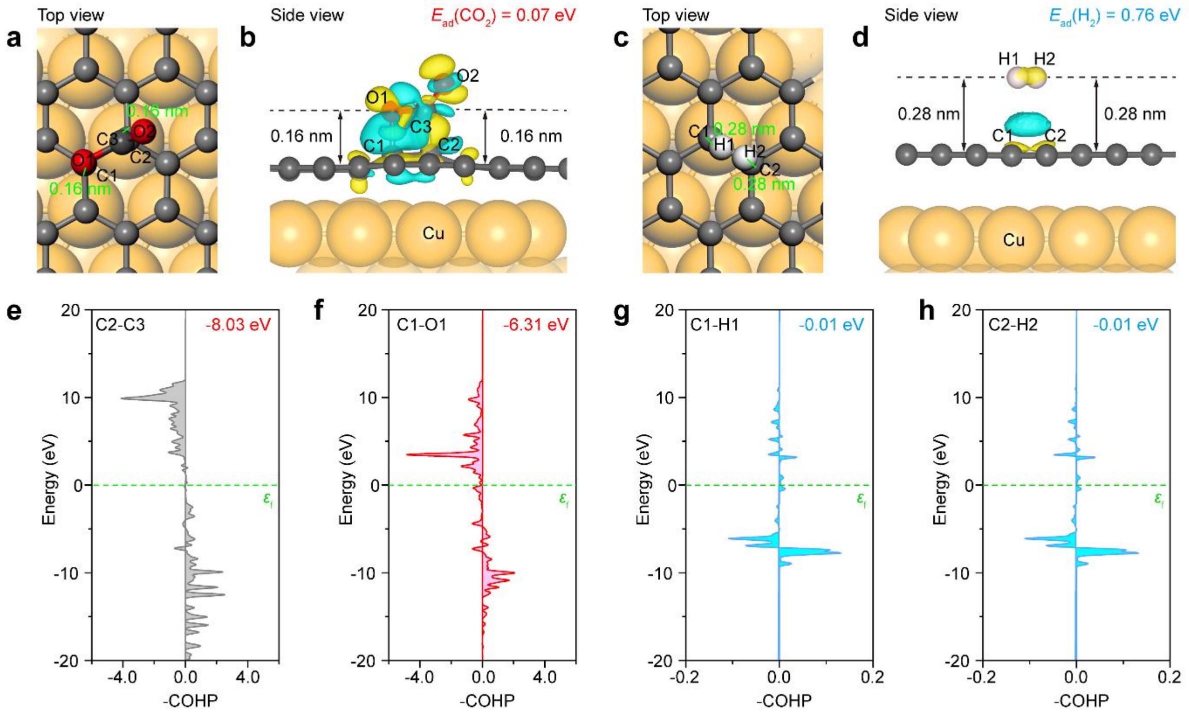

Supplementary Fig. 6. Comparison of the graphene etching ability of CO_2 and H_2. a Top view of the CO_2 molecule adsorbed on top of the perfect MLG/Cu. **b** Side view of the charge transfer between the CO_2 molecule and the MLG/Cu. Adsorption energy of CO_2 and H_2 molecules on graphene is denoted as $E_{ad}(CO_2)$ and $E_{ad}(H_2)$, respectively. Top view of the H_2 molecule adsorbed on top of the perfect MLG/Cu. **d** Side view of the charge transfer between the CO_2 molecule and the MLG/Cu. **e,f** COHP between the C (**e**) or O (**f**) atoms of the CO_2 molecule and the C atoms in the graphene lattice. **g,h** COHP

between the two H atoms of the H_2 molecule and the C atoms in the graphene lattice.

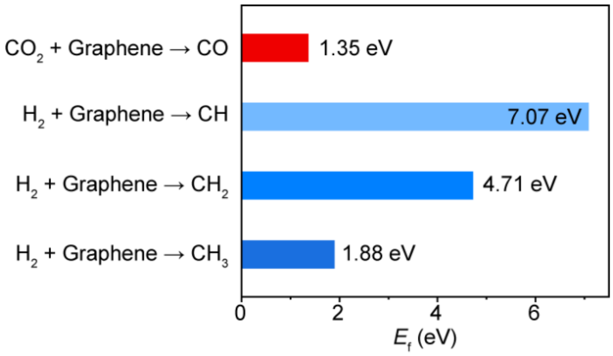

Supplementary Fig. 7. Comparison of the graphene etching ability of CO_2 and H_2 based on the formation energy (E_f) of the different reactions.

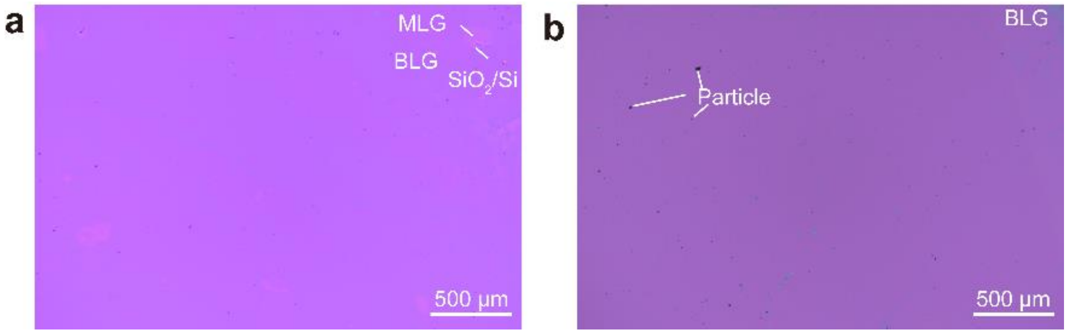

Supplementary Fig. 8. OM images of large-area bilayer graphene (BLG) transferred onto SiO_2/Si substrate. a Optical microscope (OM) image of BLG with some breakage regions, where the monolayer graphene (MLG) area is also observed. **b** OM image of the intact BLG, whose size is ~ 3 * 2 mm^2.

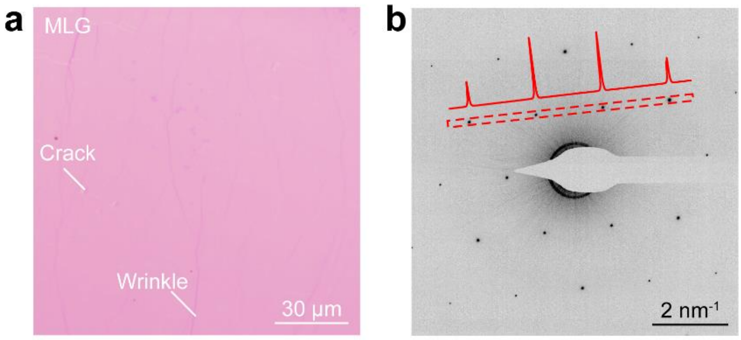

Supplementary Fig. 9. Synthesis of the monolayer graphene (MLG) without using CO_2 during the high-temperature growth stage. a Optical microscope image of the MLG transferred onto a SiO_2/Si substrate. **b** Selected area electron diffraction (SAED) pattern of the MLG transferred onto a

commercial transmission electron microscope grid. Inset: The intensity profiles of the diffraction patterns along the red dashed lines.

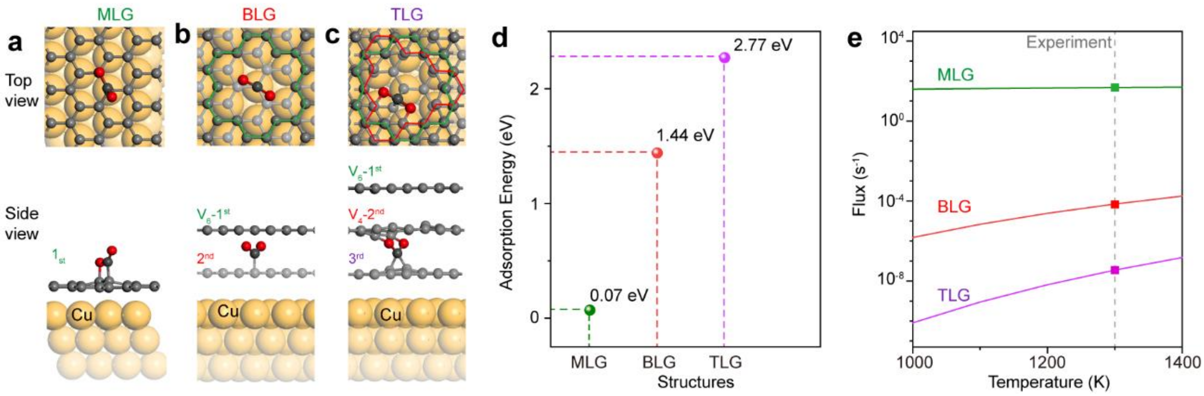

Supplementary Fig. 10. Etching ability of monolayer graphene (MLG), bilayer graphene (BLG) and tri-layer graphene (TLG) using CO_2. a-c Structures of CO_2 molecules adsorbed on MLG (**a**), BLG (**b**) and TLG (**c**) surfaces. V_4 and V_6 correspond to the vacancy in graphene lattice after losing four and six carbon atoms respectively. **d** Adsorption energy of CO_2 molecules on MLG, BLG and TLG. **e** Comparison of the etching rate of MLG, BLG and TLG using CO_2 at different temperatures.

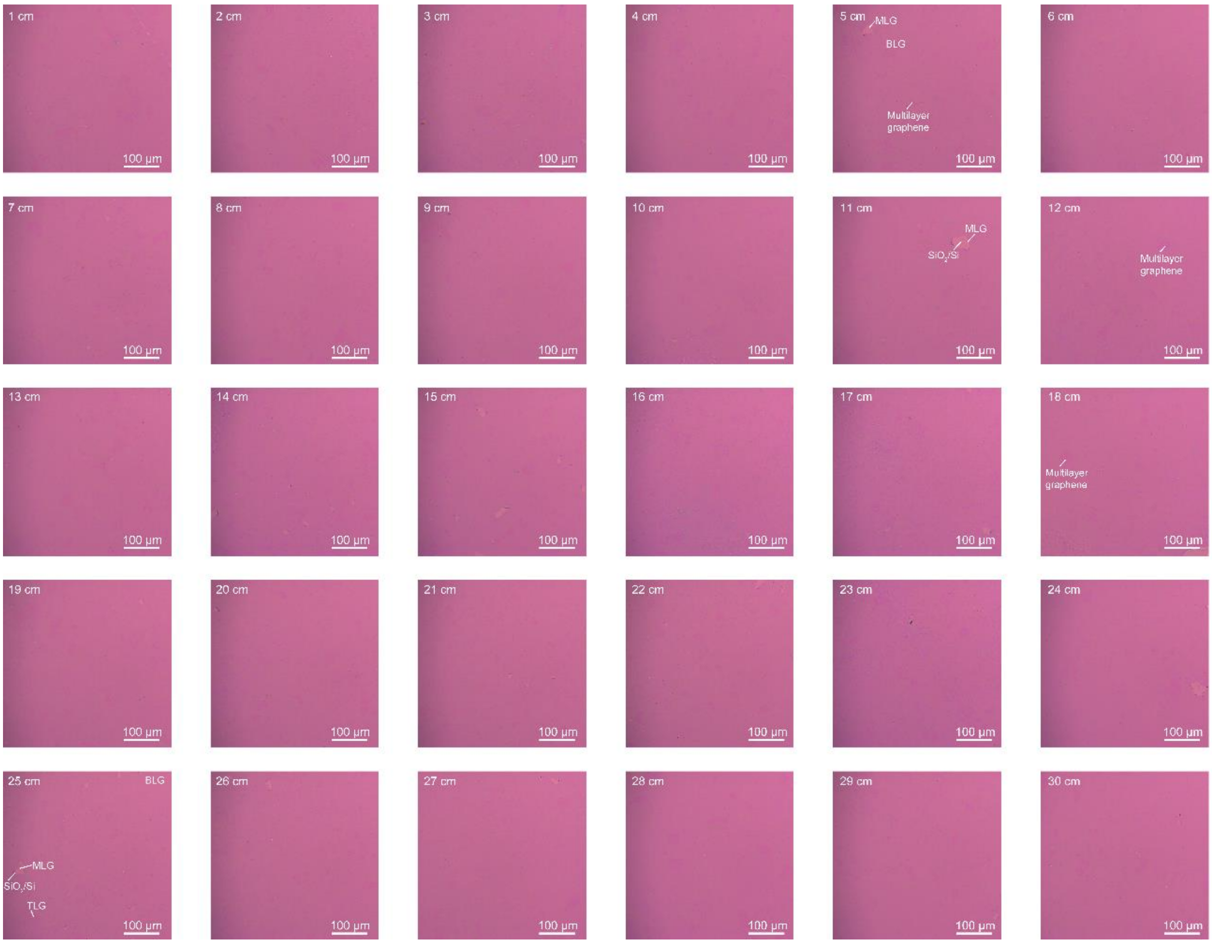

Supplementary Fig. 11. Optical microscope images of large-area bilayer graphene (BLG) grown on the polycrystalline Cu substrate acquired every 1 cm. Note that MLG and TLG are the abbreviations of monolayer graphene and tri-layer graphene, respectively.

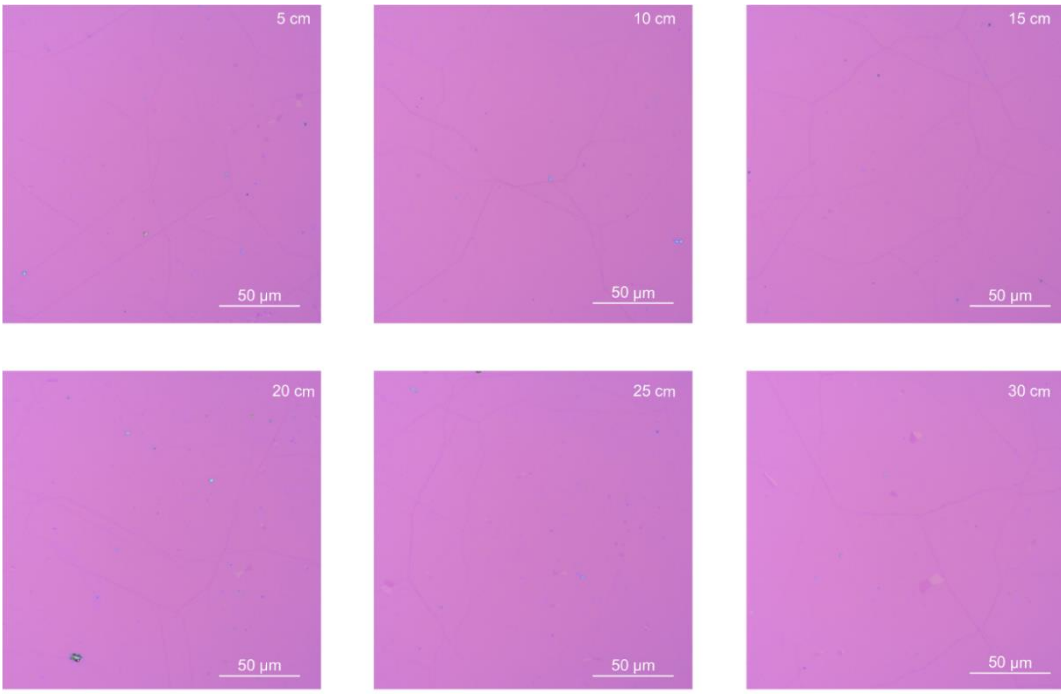

Supplementary Fig. 12. Optical microscope images of bilayer graphene film acquired using x50 objective.

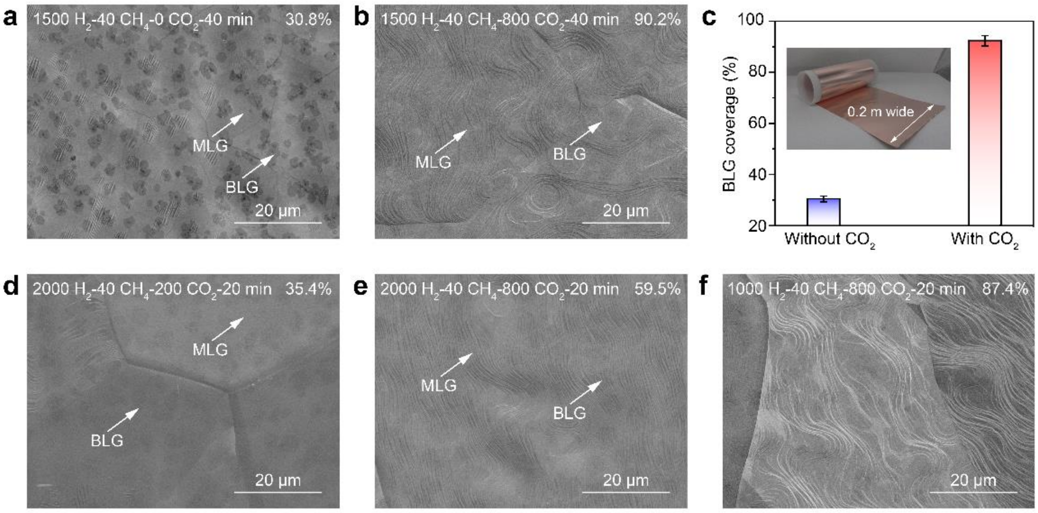

Supplementary Fig. 13. Comparison of bilayer coverage of the graphene films synthesized on large-size Cu foil (2 m * 0.2 m) inside a quartz tube with diameter of 10 inch. a-c Scanning electron microscopy images of the as-synthesized graphene without (**a**) and with (**b**) utilizing CO_2 and the corresponding statistical results (**c**). Inset in (**c**): Photograph of the meter-sized bilayer graphene (BLG) grown on the commercial Cu foil. Error bars represent standard deviations from three measurement

results for each type of sample. **d-f** Impact of the flux of CO_2 and H_2 on the bilayer coverage of graphene. MLG is the abbreviation of monolayer graphene.

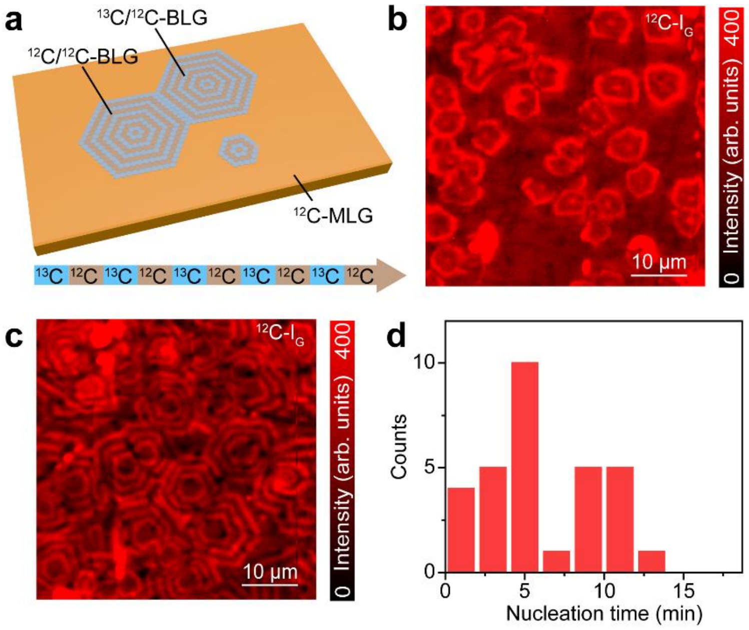

Supplementary Fig. 14. Nucleation and merging of the bilayer graphene (BLG). a Schematic of the growth of the BLG on Cu surface. **b,c** Raman mapping result of intensity of G band of the ^{12}C-graphene (^{12}C-I$_G$) with isolated bilayer domains (**b**) and the continuous BLG film (**c**). **d** Statistics of the BLG nucleation time. MLG is the abbreviation of monolayer graphene.

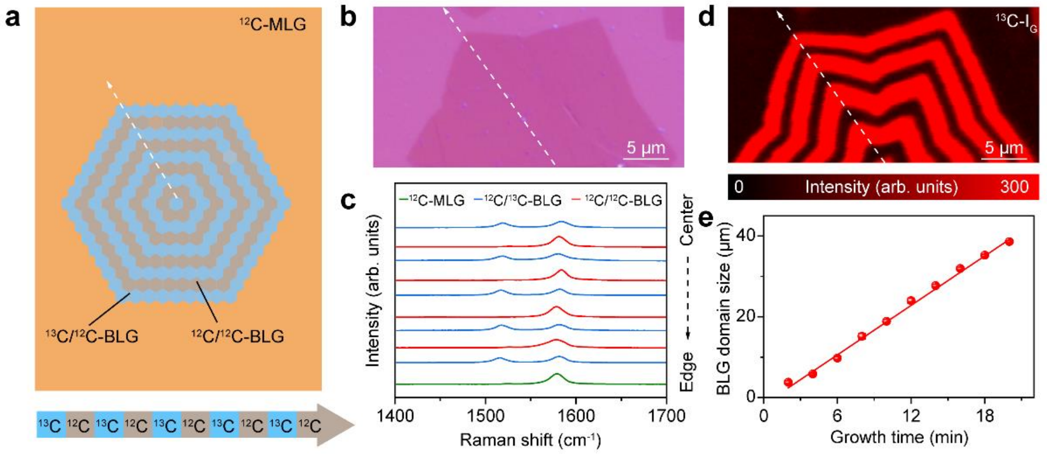

Supplementary Fig. 15. Growth dynamics of the bilayer graphene (BLG). a Schematic of the BLG growth with the aid of CO_2, via alternatively flowing $^{12}CH_4$ and $^{13}CH_4$. **b** Optical microscope image of the half BLG domain on the SiO_2/Si substrate. **c** Raman spectra of the graphene along the white dashed

line in **b**, which were acquired every 2 μm from center to the edge. Note that the green, blue, and red spectra correspond to ^{12}C-monolayer graphene (MLG), $^{13}C/^{12}C$ BLG and $^{12}C/^{12}C$ BLG, respectively. **d** Raman map result of the intensity of G band of ^{13}C-graphene (^{13}C-I_G). **e** Domain size of the BLG as a function of the growth time, based on which a linear fitting is conducted (dashed red line).

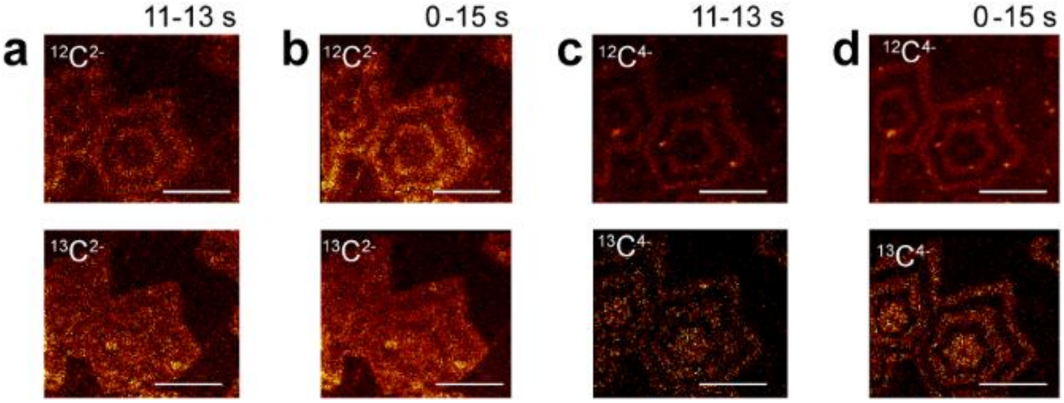

Supplementary Fig. 16. Time-of-flight secondary ion mass spectrometry (ToF-SIMS) measurement results. a,b Spatial distribution of $^{12}C^{2-}$ (top) and $^{13}C^{2-}$ (bottom) in the scanned regions during the sputter time of 11-13 s **(a)** and 0-15 s **(b). c,d** Spatial distribution of $^{12}C^{4-}$ (top) and $^{13}C^{4-}$ (bottom) in the scanned regions during the sputter time of 11-13 s (**c**) and 0-15 s **(d).** Scale bar: 5 μm.

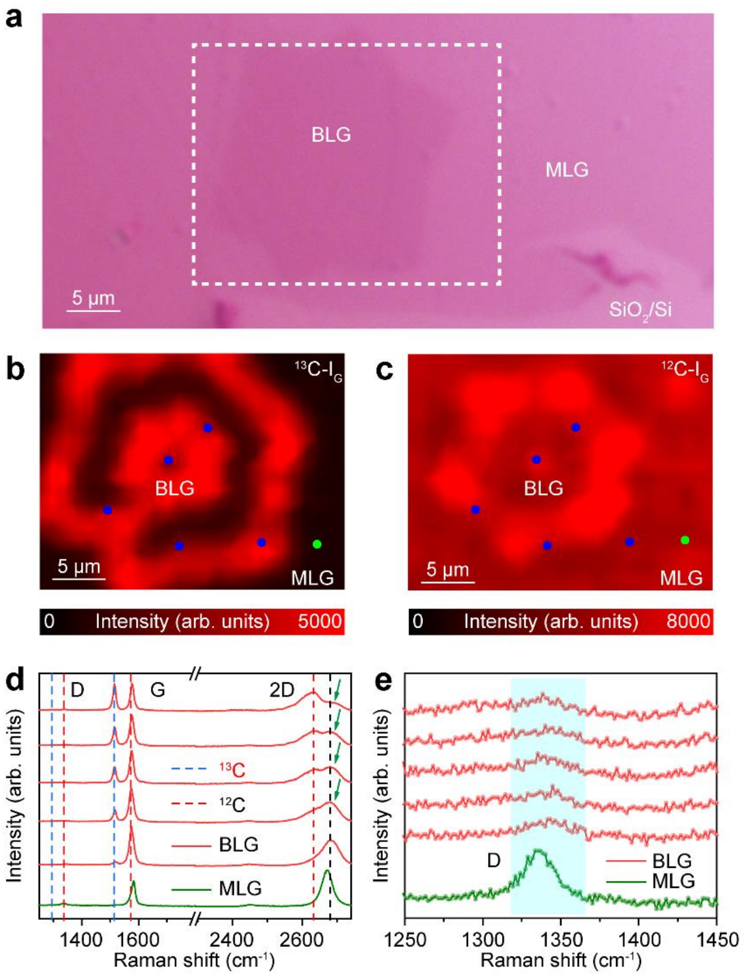

Supplementary Fig. 17. Confirmation of the bilayer graphene (BLG) stacking order. a OM image of the BLG domain after the mild oxygen plasma treatment to create point defects in the top graphene layer. **b,c** Raman map results of intensity of G bands of ^{13}C-graphene (^{3}C-I_G) (**b**) and ^{12}C-graphene (^{12}C-I_G) (**c**). **d** Raman spectra of the ^{12}C-monolayer graphene (MLG) (green) and ^{12}C/^{13}C-BLG (red) after plasma treatment. **e** Raman D bands appeared in ^{12}C-MLG (green) and ^{12}C/^{13}C-BLG (red) regions after plasma treatment.

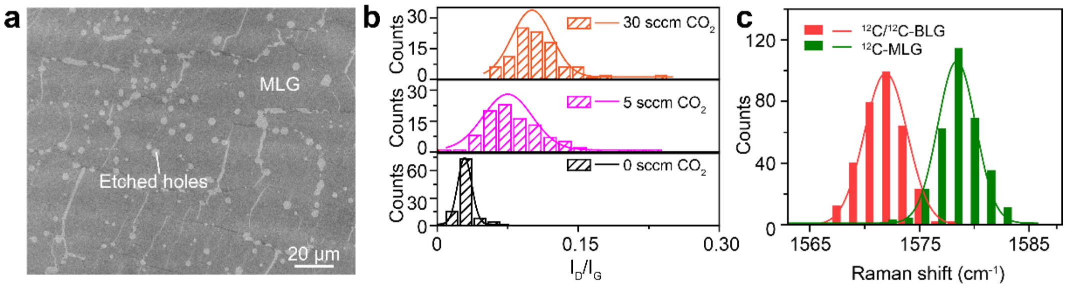

Supplementary Fig. 18. Formation and repairing of defects in graphene before and after the bilayer graphene growth. a SEM image of the monolayer graphene (MLG) on Cu surface after mild CO_2 etching (10 sccm CO_2, 1 min). **b** Statistical histogram of the defect density in the MLG treated with different CO_2 fluxes based on the intensity ratios of 2D to G bands (I_{2D}/I_G). **c** Comparison of the positions of the G bands in the ^{12}C-MLG (green) and the ^{12}C/^{12}C BLG (red).

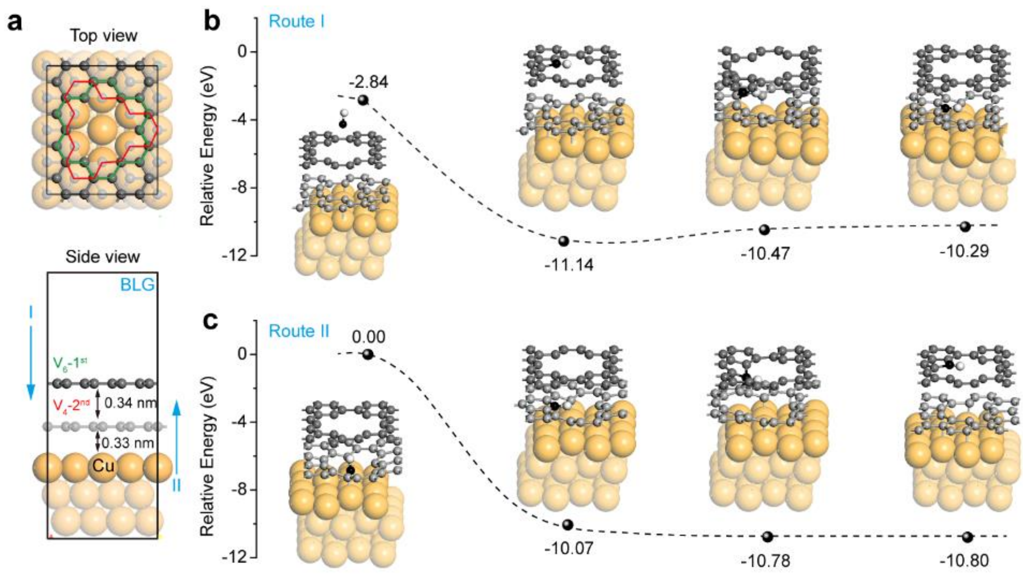

Supplementary Fig. 19. Self-healing of defects in bilayer graphene (BLG) at high temperature on Cu substrate. a Structure of the defective graphene on Cu surface. **b** Healing of graphene via the supply of extra active carbon species from the gas phase. **c** Heal of graphene via the supply of extra active carbon species from the Cu substrate.

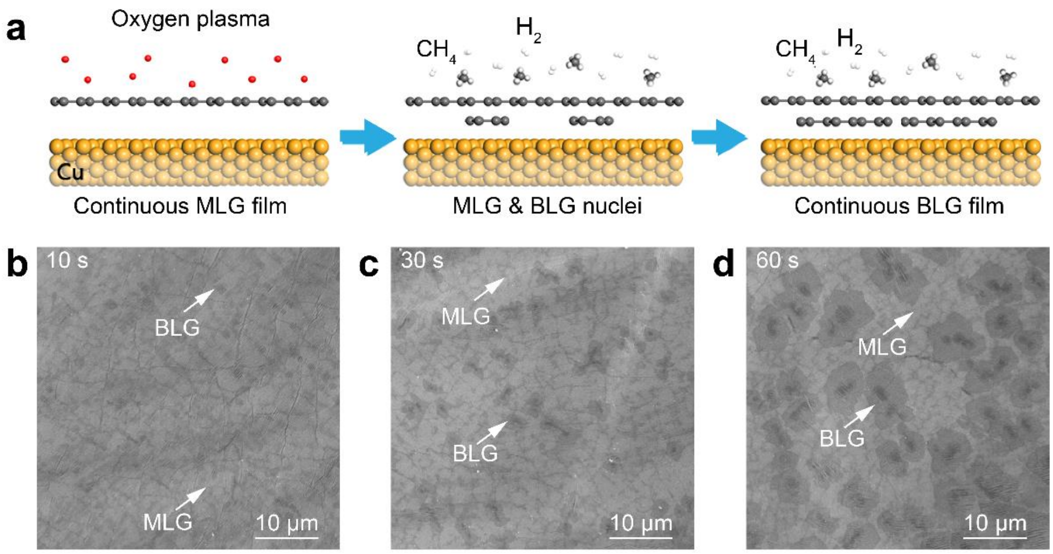

Supplementary Fig. 20. Impact of defect density in the monolayer graphene (MLG) on the growth of bilayer graphene (BLG) on Cu. a Schematic of the BLG growth on a Cu substrate after the formation of point defects in the upmost graphene layer. **b-d** Scanning electron microscope images of graphene films with varied BLG coverage after etching of the MLG using oxygen plasma for 10 s (**b**), 30 s (**c**), and 60 s (**d**).

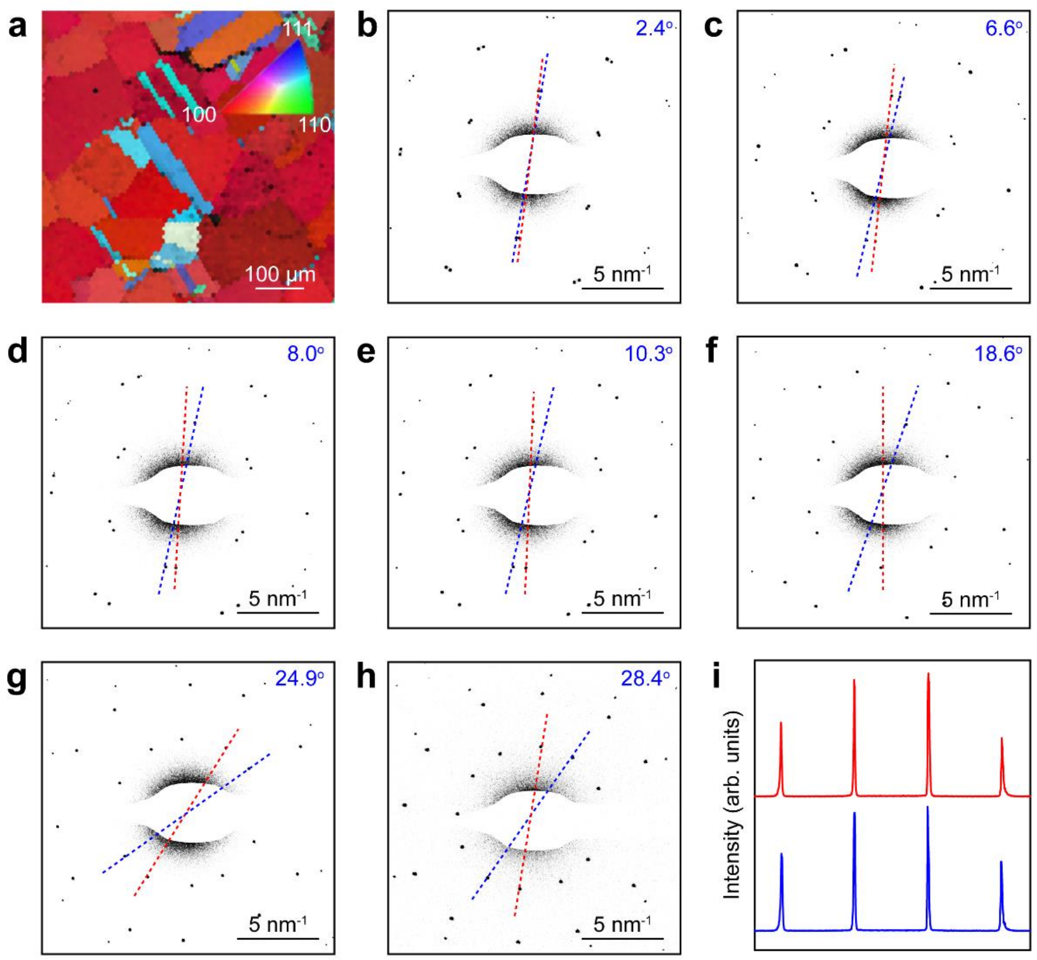

Supplementary Fig. 21. Stacking order of the bilayer graphene grown on the Cu(100)-dominated polycrystalline Cu foil. a Electron back-scattered diffraction (EBSD) image of polycrystalline Cu after graphene growth. **b-h** Selected area electron diffraction (SAED) patterns of the non-AB stacking BLG (tBLG). The twist angles are acquired based on the intersection angles of the dashed red and blue lines in each SEAD pattern. **i** Intensity profiles along the red (top) and blue (bottom) dashed lines in (**b**).

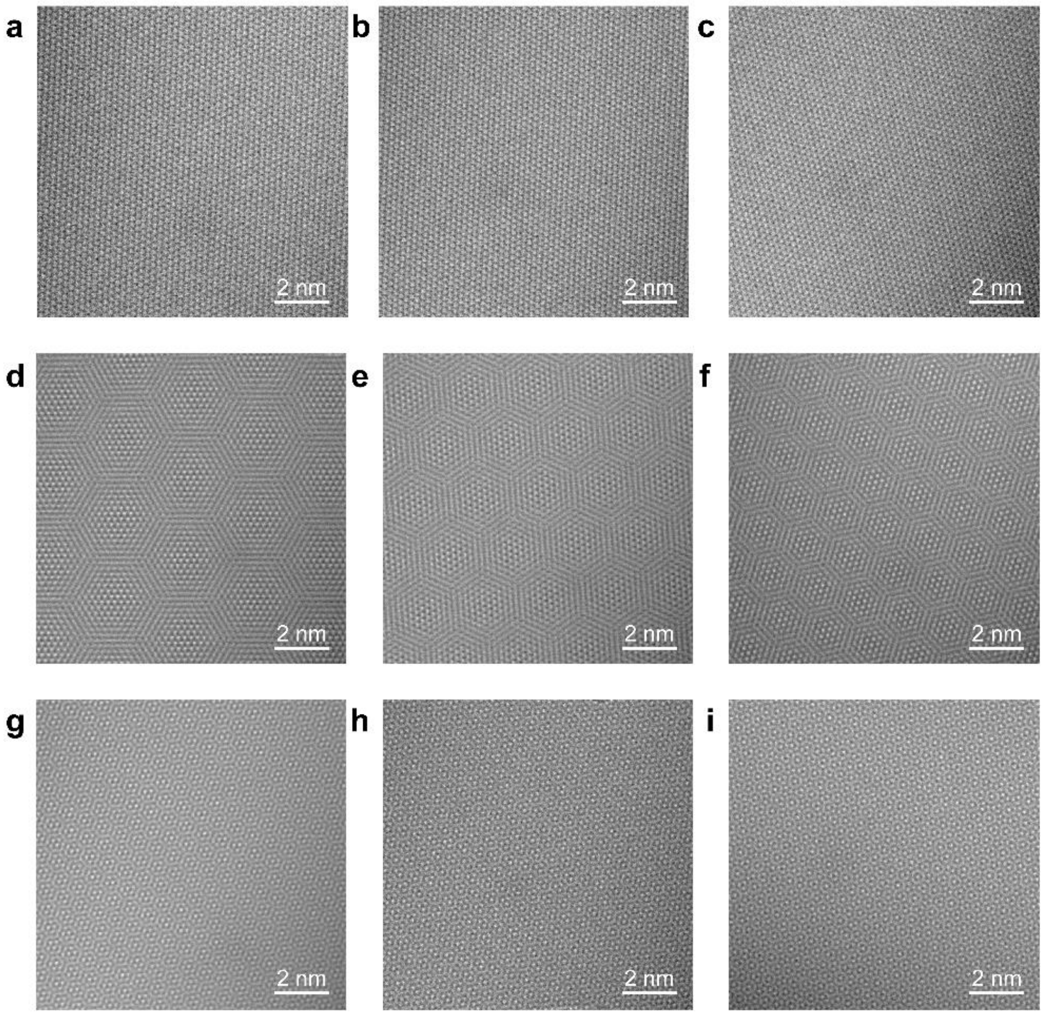

Supplementary Fig. 22. High-resolution transmission electron microscope (HRTEM) images of the BLG. a-i HRTEM images of the AB-stacking BLG (AB-BLG) (**a-c**) and the non-AB-stacking BLG (tBLG) (**d-i**).

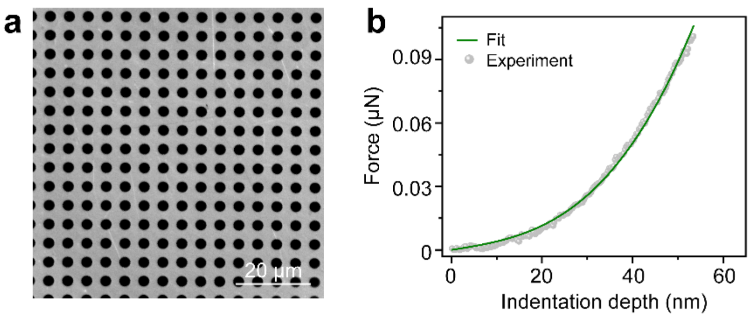

Supplementary Fig. 23. Mechanical property measurement of the suspended bilayer graphene (BLG). a Scanning electron microscope image of the suspended BLG on transmission electron

microscope (TEM) grids, where the negligible breakage indicates the enhanced mechanical strength of the BLG. **b** AFM nano-indentation measurement result of the monolayer graphene (MLG).

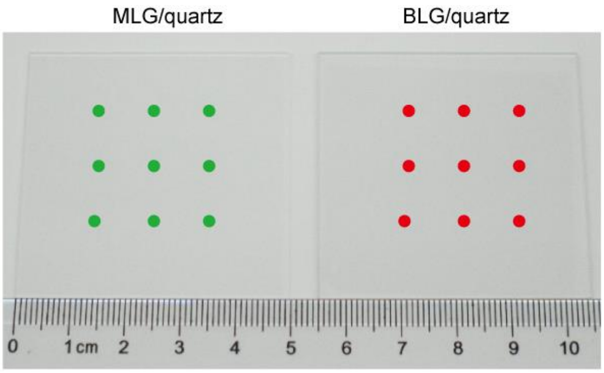

Supplementary Fig. 24. Photograph of the monolayer graphene (MLG) (left) and bilayer graphene (BLG) (right) transferred to quartz substrates for transmittance measurement in large area.

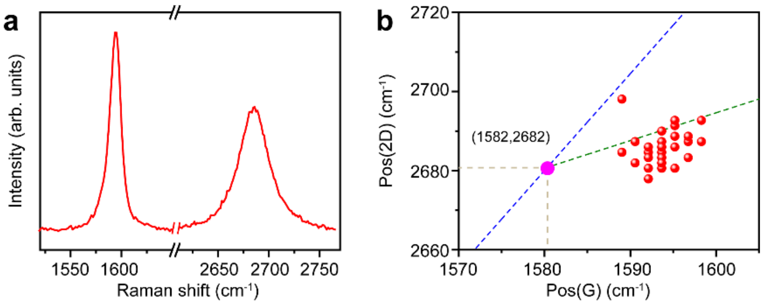

Supplementary Fig. 25. Doping analysis of the transferred bilayer graphene (BLG) using Raman. **a** Raman spectrum of the BLG transferred on SiO_2/Si for sheet resistance measurement. **b** Statistical result showing the relationship between the peak position of G and 2D bands (Pos(G) and Pos(2D)), in which the dashed blue line corresponds to the strain-free regions with slope of 2.45 while the dashed green line corresponds to the doping-free regions for monolayer graphene (MLG), which is plotted as a guidance here.

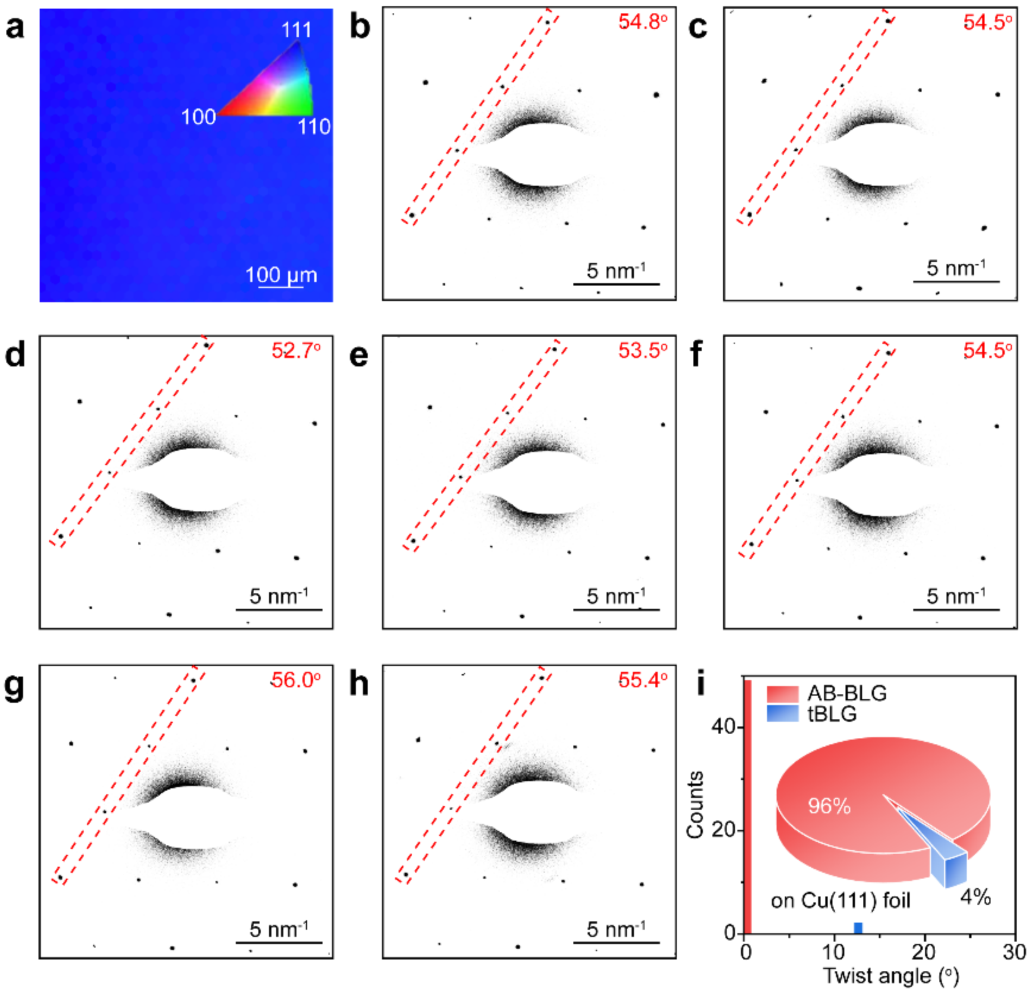

Supplementary Fig. 26. Stacking order of the bilayer graphene (BLG) grown on single-crystal Cu(111) foil. a Electron back-scattered diffraction (EBSD) image of Cu(111) substrate after the AB stacking BLG (AB-BLG) growth. **b-h** Selected area electron diffraction (SAED) patterns of the AB-BLG. **i** Distribution of twist angles and the corresponding statistical results of the stacking order (AB stacking (red color) or non-AB stacking (tBLG, blue color)) based on SAED patterns of the BLG grown on the single-crystal Cu(111) foil substrate.

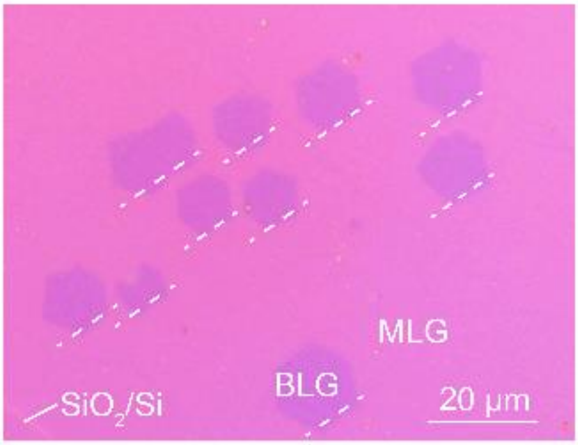

Supplementary Fig. 27. Optical microscope image of the isolated BLG domains grown on Cu(111) foil.

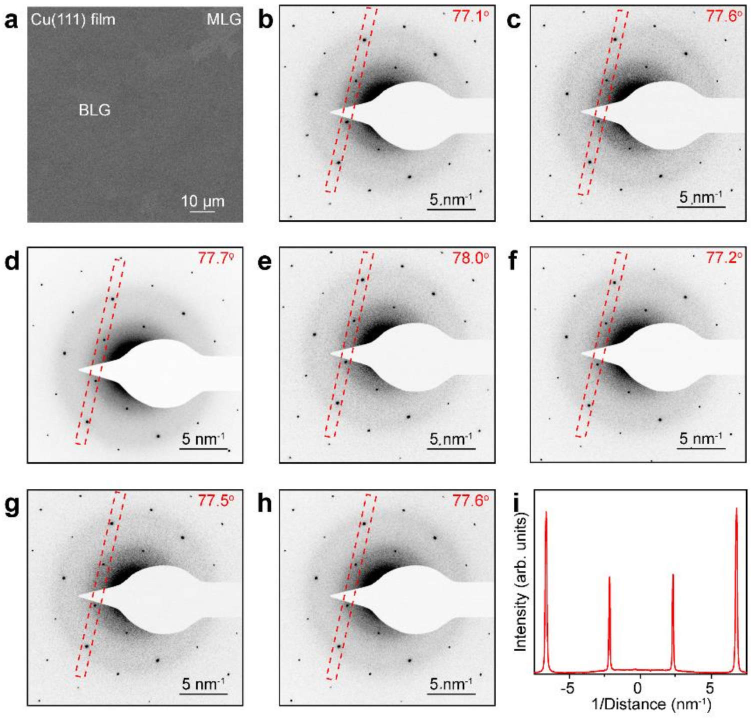

Supplementary Fig. 28. Stacking order of the bilayer graphene (BLG) grown on single-crystal Cu(111) film. a Scanning electron microscope image of the BLG grown on a Cu(111) film substrate. **b-h** Selected area electron diffraction patterns of the AB stacking BLG. (I) Intensity profile along the red dashed line in (**b**). MLG is the abbreviation of monolayer graphene.

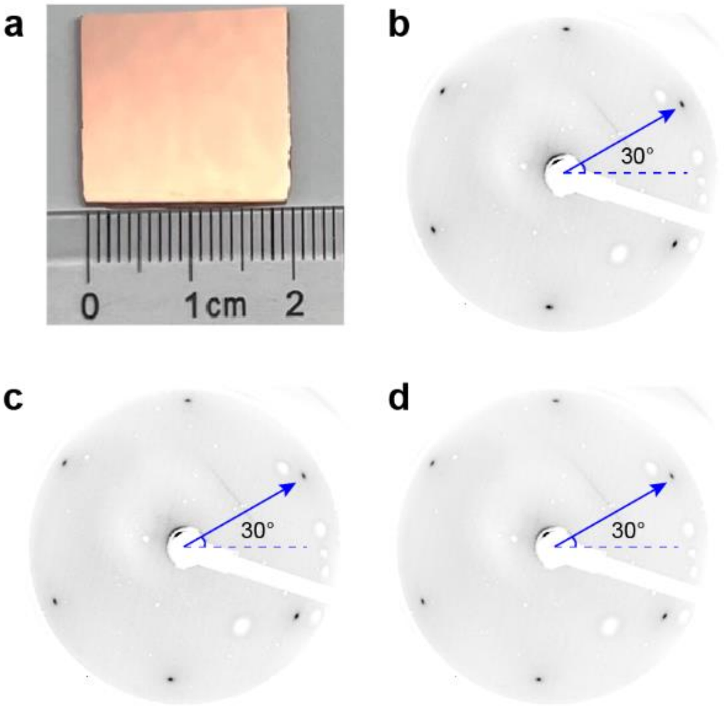

Supplementary Fig. 29. Low energy electron diffraction (LEED) patterns of the AB-stacking bilayer graphene (AB-BLG) film grown on the Cu(111)/sapphire substrate. a Photograph of the cm-sized AB-BLG/Cu(111)/sapphire sample. **b-d** LEED patterns acquired on the AB-BLG/Cu(111) sample. The blue arrows denote the orientation of the BLG film in different positions.

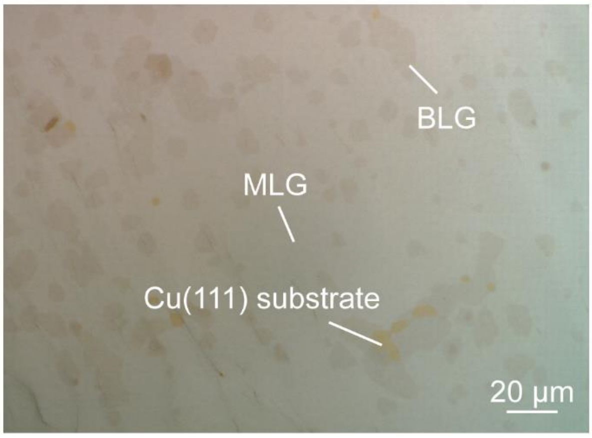

Supplementary Fig. 30. Optical microscope image of the isolated bilayer graphene (BLG) domains grown on Cu(111) film. MLG is the abbreviation of monolayer graphene.

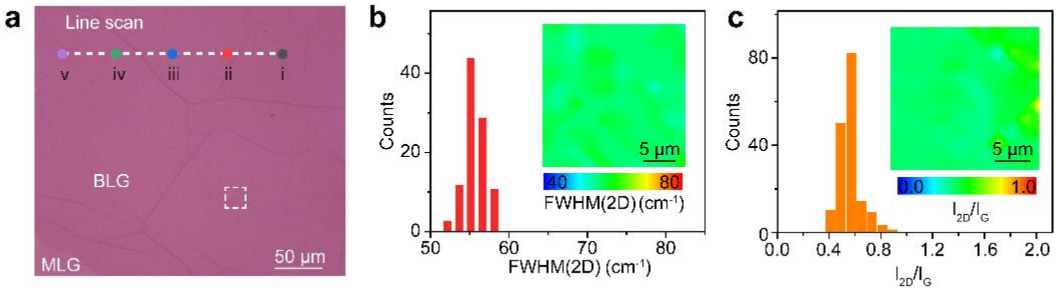

Supplementary Fig. 31. Optical microscope (OM) and Raman characterization of the AB stacking BLG (AB-BLG). **a** OM image of the AB-BLG transferred to a SiO_2/Si substrate. **b** Statistical results of the full width ad half maximum of 2D band (FWHM(2D)) of the AB-BLG. Inset: Mapping result of FWHM(2D). **c** Statistical histogram of the intensity ratio of 2D to G bands (I_{2D}/I_G). Inset: Mapping result of I_{2D}/I_G.

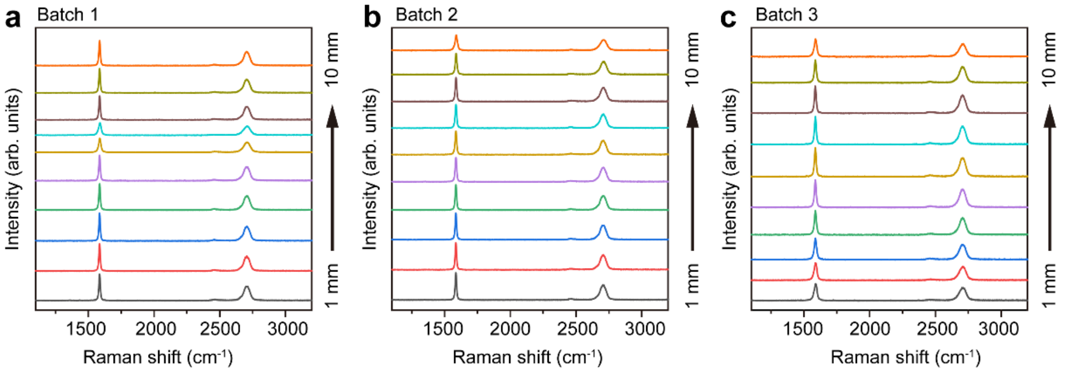

Supplementary Fig. 32. Raman characterization of the AB stacking bilayer graphene (AB-BLG) samples. **a** Sample 1. **b** Sample 2. **c** Sample **3.**

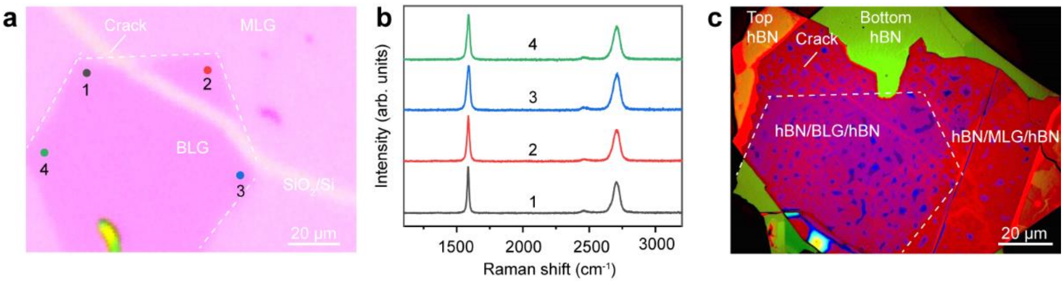

Supplementary Fig. 33. Optical microscope (OM) and Raman characterization of the AB stacking bilayer graphene (AB-BLG) domain used to fabricate the dual-gate device. **a** OM image of the AB-BLG domain transferred on a SiO_2/Si substrate. **b** Raman spectra acquired on four different positions marked in (**a**). **c** OM image of the hBN/BLG/hBN sandwiched structure. MLG is abbreviated for

monolayer graphene.

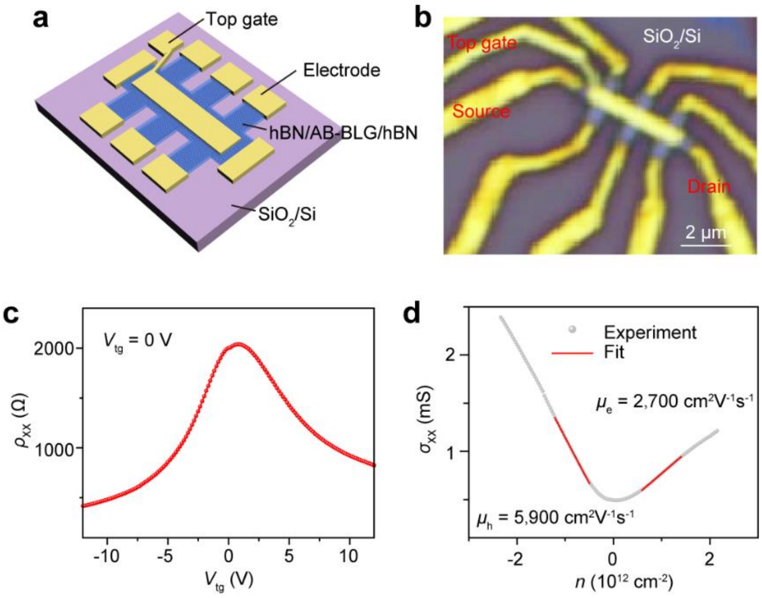

Supplementary Fig. 34. Electrical measurement of the dual gate AB-stacking bilayer graphene (AB-BLG) Hall bar device. **a** Schematic of the Hall bar device of the AB-BLG. **b** OM image of the AB-BLG dual gate Hall bar device. **c** Resistivity (ρ_{xx}) of the AB-BLG as a function of the top gate voltage (V_{tg}), which is measured at room temperature (300 K) with zero back gate voltage (V_{bg}). **d** Conductivity (σ_{xx}) of the AB-BLG as a function of the carrier concentration (n).

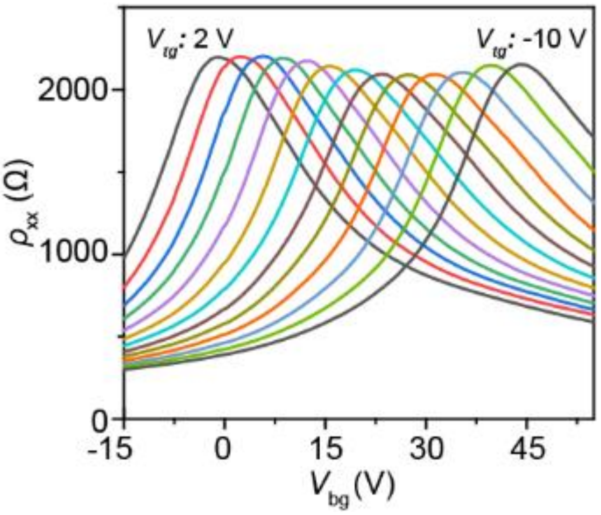

Supplementary Fig. 35. Transfer curves of the dual gate AB-BLG Hall bar device with varied top gate voltage (V_{tg})and back gate voltage (V_{bg}).

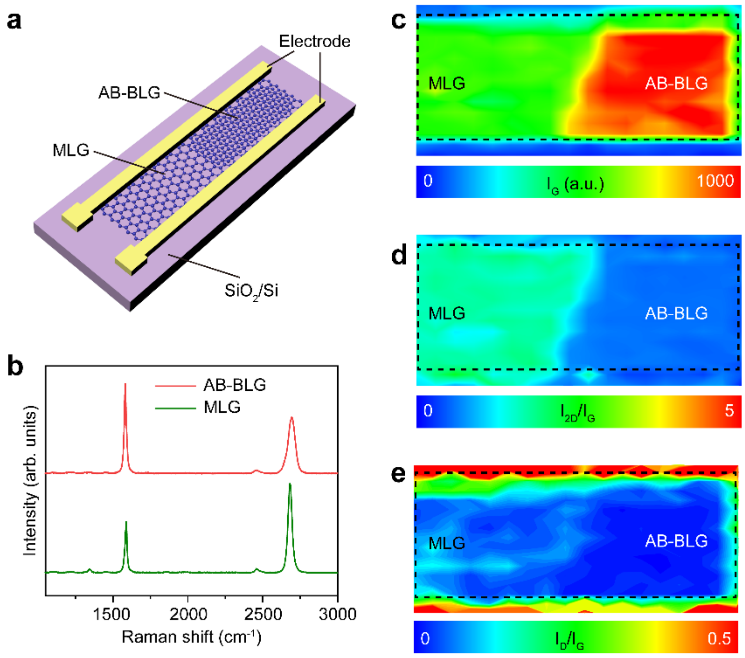

Supplementary Fig. 36. Raman characterization of the graphene-based photodetector. a Schematic of the graphene-based photodetector composed of monolayer graphene (MLG) and AB stacking bilayer graphene (AB-BLG). **b** Raman spectra of the MLG (green) and AB-BLG (red). **c-e** Raman mapping results of the intensity of G band (I_G)(**c**), intensity ratio of 2D to G bands (I_{2D}/I_G) (**d**), and intensity ratio of D to G bands (I_D/I_G) (**e**), with the MLG in the left and the AB-BLG in the right.

Supplementary Table

Supplementary Table 1. Statistic of the bilayer coverage and domain size of the BLG grown on Cu[9,33-59] Note that for clear comparison, only those with BLG growth time shorter than 200 min are plotted in Fig. 1g, while the others are only listed in the Supplementary Table 1.

Year	Group	BLG coverage (%)	BLG domain size (μm)	Growth time (min)	Fig.s	Reference
2011	Kaustav Banerjee	19	<5	20	Fig. 5	Carbon, 2011, 49, 4122-4130[33]
2011	Zhongfan Liu	67	30	120	Fig. 1	Nano Lett., 2011, 11, 1106–1110[34]
2012	Ladislav Kavan	4	<5	6	Fig. 1	Carbon, 2012, 50, 3682-3687[35]
2012	Jianguo Hou	73	<5	30	Fig. 2	J. Phys. Chem. C, 2012, 116, 10557−10562[36]
2012	Bruce H. Weiller	75	<5	20	Fig. 2	Small, 2012, 9, 1415-1422[37]
2012	Xiangfeng Duan	40/99	<40 um	60/180	Supplementary Fig. 1, Fig. 3	ACS Nano, 2012, 9, 8241-8249[38]
2013	Jing Kong	61	5-10	180	Fig. 1	Nano Lett., 2013, 13, 1541−1548[9]
2014	Richard Martel	61	<10	50	Fig. 4	J. Phys. Chem. C, 2014, 118, 21532−21540[39]
2014	Weiwei Cai	78	~10	360	Fig. 4	Small, 2014, 12, 1418–1422[40]
2014	Li-Jun Wan	90	<5	210	Supplementary Fig. 2	Chem. Commun., 2014, 50, 11012-11015[41]
2014	Shigeo Maruyama	94	<10	90	Fig. 2	ACS Nano, 2014, 11, 11631-11638[42]
2014	Jing Kong	8/40/86/96	20-30	5/30/60/120	Fig. 2	ACS Nano, 2014, 6, 6491-6499[43]

2015	Zhengtang Luo	84	22-63	120	Fig. 3	Nanoscale, 2015, 7, 2391-2399[44]
2015	Young Jae Song	97	10-50	720	Fig. 2	Nanoscale, 2015, 7, 10357-10361[45]
2016	Rodney S. Ruoff	56	300-500	120	Fig. 1	Nat. Nanotechnol., 2016, 11, 426-431[46]
2016	Hongtao Wang	~90	<5	180	Fig. 3	Nanoscale, 2016, 8, 20001-20007[47]
2016	Gui Yu	93	<20	45	Fig. 2	J. Mater. Chem. C, 2016, 4, 7464-7471[48]
2016	Jong-Souk Yeo	100	<5	70	Fig. 1	Carbon, 2016, 105, 205-213[49]
2017	Hyung Gyu Park	37	~2	60	Fig. 3	2D Mater., 2017, 4, 035023[50]
2018	Hyesung Park	22	3-10	30	Fig. 3	J. Nanomaterials, 2018, 146, 549-556[51]
2018	Wei-Yen Woon	30	120	120	Fig. 2	Carbon, 2018, 135, 118-124[52]
2018	Xian Zhao	41	~3	15	Fig. 8	J. Mater. Sci. Mater. Elect., 2018, 29, 4495–4502[53]
2018	Hengxing Ji	77	<20	30	Fig. 2	Chem. Mater. 2018, 30, 7852-7859[54]
2019	Michael A Cullinan	50	<5	30	Fig. 2	Nanotechnology, 2019, 30, 235602[55]
2019	Xuesong Li	56	10-20	90	Fig. 2	J. Materiomics, 2019, 5, 463-470[56]
2019	Zhengzong Sun	95	10-20	180	Fig. 2	Chem. Mater., 2019, 31, 6105−6109[57]
2020	Wei-Yen Woon	50	<40	70	Fig. 2	Nanotechnology, 2020, 31, 435603[58]
2020	Hongwei Zhu	76	<30	60	Fig. 1	Sci. China Mater., 2020, 63, 1973-1982[59]

2022	Zhengzong Sun	65	~100	40	Fig. S12	*ACS Cent. Sci*. 2022, 8, 3, 394–401[7]

Supplementary References

1. Yang, X. et al. Insight into CO_2 etching behavior for efficiently nanosizing graphene. *Adv. Mater. Interfaces* **4**, 1601065 (2017).

2. Xie, L., Jiao, L., Dai, H. Selective etching of graphene edges by hydrogen plasma. *J. Am. Chem. Soc.* **132**, 14751-14753 (2010).

3. Zhang, Y., Li, Z., Kim, P., Zhang, L., Zhou, C. Anisotropic hydrogen etching of chemical vapor deposited graphene. *ACS Nano* **6**, 126-132 (2012).

4. Ma, T. et al. Repeated growth–etching–regrowth for large-area defect-free single-crystal graphene by chemical vapor deposition. *ACS Nano* **8**, 12806-12813 (2014).

5. Zhang, J. et al. Large-Area synthesis of superclean graphene via selective etching of amorphous carbon with carbon dioxide. *Angew. Chem. Int. Ed. Engl.* **58**, 14446-14451 (2019).

6. Yao, W. et al. Bottom-up-etching-mediated synthesis of large-scale pure monolayer graphene on cyclic-polishing-annealed Cu (111). *Adv. Mater.* **34**, 2108608 (2022).

7. Gong, P. et al. Precise CO_2 reduction for bilayer graphene. *ACS Cent. Sci.* **8**, 394-401 (2022).

8. Xu, X. et al. Ultrafast growth of single-crystal graphene assisted by a continuous oxygen supply. *Nat. Nanotechnol.* **11**, 930-935 (2016).

9. Fang, W. et al. Rapid identification of stacking orientation in isotopically labeled chemical-vapor grown bilayer graphene by Raman spectroscopy. *Nano Lett.* **13**, 1541-1548 (2013).

10. Li, Q. et al. Growth of adlayer graphene on Cu studied by carbon isotope labeling. *Nano Lett.* **13**, 486-490 (2013).

11. Zheng, L. et al. Robust ultraclean atomically thin membranes for atomic-resolution electron microscopy. *Nat. Commun.* **11**, 541 (2020).

12. Wu, P., Zhai, X., Li, Z., Yang, J. Bilayer Graphene growth via a penetration mechanism. *J. Phys. Chem. C* **118**, 6201-6206 (2014).

13. Chen, J. et al. Self healing of defected graphene. *Appl. Phys. Lett.* **102**, 103107 (2013).

14. Vicarelli, L., Heerema, S. J., Dekker, C., Zandbergen, H. W. Controlling defects in graphene for optimizing the electrical properties of graphene nanodevices. *ACS Nano* **9**, 3428-3435 (2015).

15. Wang, L., Zhang, X., Chan, H. L., Yan, F., Ding, F. Formation and healing of vacancies in graphene chemical vapor deposition (CVD) growth. *J. Am. Chem. Soc.* **135**, 4476-4482 (2013).

16. Higgins, M. J. et al. Noninvasive determination of optical lever sensitivity in atomic force microscopy. *Rev. Sci. Instrum.* **77**, 013701 (2006).

17. Lee, C., Wei, X., Kysar, J. W., Hone, J. Measurement of the elastic properties and intrinsic strength of monolayer graphene. *Science* **321**, 385-388 (2008).

18. Ould, NE. M., Boujnah, M., Benyoussef, A., Kenz, A. E. Electronic and electrical conductivity of AB and AA-stacked bilayer graphene with tunable layer separation. *J. Supercond. Nov. Magn.* **30**, 1263-1267 (2017).

19. Li, X. et al. Transfer of large-area graphene films for high-performance transparent conductive electrodes. *Nano Lett.* **9**, 4359-4363 (2009).

20. Bae, S. et al. Roll-to-roll production of 30-inch graphene films for transparent electrodes. *Nature Nanotechnol.* **5**, 574-578 (2010).

21. Wang, Y., Tong, S. W., Xu, X. F., Özyilmaz, B., Loh, K. P. Interface engineering of layer-by-layer stacked

graphene anodes for high-performance organic solar cells. *Adv. Mater.* **23**, 1514-1518 (2011).
22. Lin Y, et al. Graphene/semiconductor heterojunction solar cells with modulated antireflection and graphene work function. *Energy Environ. Sci.* **6**, 108-115 (2013).
23. Sui Y, Appenzeller J. Screening and interlayer coupling in multilayer graphene field-effect transistors. *Nano Lett.* **9**, 2973-2977 (2009).
24. Liu N, Zhou S, Zhao J. Electrical conductance of graphene with point defects. *Acta Phys-Chim Sin* **35**, 1000-6818 (2019).
25. Araki, Y. et al. Twist angle-dependent molecular intercalation and sheet resistance in bilayer graphene. *ACS Nano* **16**, 14075-14085 (2022).
26. Lee JE, Ahn G, Shim J, Lee YS, Ryu S. Optical separation of mechanical strain from charge doping in graphene. Nat. Commun. 3, 1-8 (2012).
27. Choi W, Shehzad MA, Park S, Seo Y. Influence of removing PMMA residues on surface of CVD graphene using a contact-mode atomic force microscope. RSC Adv. 7, 6943-6949 (2017).
28. Sun L, et al. Toward epitaxial growth of misorientation-free graphene on Cu (111) foils. *ACS Nano* **16**, 285-294 (2021).
29. Deng B, et al. Wrinkle-free single-crystal graphene wafer grown on strain-engineered substrates. *ACS Nano* **11**, 12337-12345 (2017).
30. Grebenko, A. K. et al. High-quality graphene using boudouard reaction. *Adv. Sci.* **9**, 2200217 (2022).
31. Nguyen, V. L. et al. Seamless stitching of graphene domains on polished copper (111) foil. *Adv. Mater.* **27**, 1376-1382 (2015).
32. Ma, W, et al. Interlayer epitaxy of wafer-scale high-quality uniform AB-stacked bilayer graphene films on liquid Pt_3Si/solid Pt. *Nat. Commun.* **10**, 2809 (2019).
33. Liu, W., Li, H., Xu, C., Khatami, Y., Banerjee, K. Synthesis of high-quality monolayer and bilayer graphene on copper using chemical vapor deposition. *Carbon* **49**, 4122-4130 (2011).
34. Yan, K., Peng, H., Zhou, Y., Li, H., Liu, Z. Formation of bilayer bernal graphene: layer-by-layer epitaxy via chemical vapor deposition. *Nano Lett.* **11**, 1106-1110 (2011).
35. Kalbac, M., Frank, O., Kavan, L. The control of graphene double-layer formation in copper-catalyzed chemical vapor deposition. *Carbon* **50**, 3682-3687 (2012).
36. Li, Z. et al. Graphene thickness control via gas-phase dynamics in chemical vapor deposition. *J. Phys. Chem. C* **116**, 10557-10562 (2012).
37. Wassei, J. K. et al. Chemical vapor deposition of graphene on copper from methane, ethane and propane: evidence for bilayer selectivity. *Small* **8**, 1415-1422 (2012).
38. Liu, L. et al. High-yield chemical vapor deposition growth of high-quality large-area AB-stacked bilayer graphene. *ACS Nano* **6**, 8241-8249 (2012).
39. Choubak, S., Levesque, P. L., Gaufres, E., Biron, M., Desjardins, P., Martel, R. Graphene CVD: Interplay between growth and etching on morphology and stacking by hydrogen and oxidizing impurities. *J. Phys. Chem. C* **118**, 21532-21540 (2014).
40. Zhao, Z. et al. Study on the diffusion mechanism of graphene grown on copper pockets. *Small* **11**, 1418-1422 (2015).
41. Li, J. et al. Controllable atmospheric pressure growth of mono-layer, bi-layer and tri-layer graphene.

ChemComm **50**, 11012-11015 (2014).

42. Zhao, P. et al. Equilibrium chemical vapor deposition growth of Bernal-stacked bilayer graphene. *ACS Nano* **8**, 11631-11638 (2014).
43. Fang, W. et al. Asymmetric growth of bilayer graphene on copper enclosures using low-pressure chemical vapor deposition. *ACS Nano* **8**, 6491-6499 (2014).
44. Gan, L. et al. Grain size control in the fabrication of large single-crystal bilayer graphene structures. *Nanoscale* **7**, 2391-2399 (2015).
45. Wu, Q. et al. Controllable poly-crystalline bilayered and multilayered graphene film growth by reciprocal chemical vapor deposition. *Nanoscale* **7**, 10357-10361 (2015).
46. Hao, Y. et al. Oxygen-activated growth and bandgap tunability of large single-crystal bilayer graphene. *Nat. Nanotechnol.* **11**, 426-431 (2016).
47. Song, Y. et al. Epitaxial nucleation of CVD bilayer graphene on copper. *Nanoscale* **8**, 20001-20007 (2016).
48. Luo, B. et al. Chemical vapor deposition of bilayer graphene with layer-resolved growth through dynamic pressure control. *J. Mater. Chem. C* **4**, 7464-7471 (2016).
49. Han, J., Lee, J-Y., Yeo, J-S.. Large-area layer-by-layer controlled and fully bernal stacked synthesis of graphene. *Carbon* **105**, 205-213 (2016).
50. Yang, N., Choi, K., Robertson, J., Park, H. G. Layer-selective synthesis of bilayer graphene via chemical vapor deposition. *2D Mater.* **4**, 035023 (2017).
51. Lee, J., Seo, J., Jung, S., Park, K., Park, H. Unveiling the direct correlation between the CVD-Grown graphene and the growth template. *J. Nanomater.* **2018**, 7610409 (2018).
52. Chan, C-C., Chung, W-L., Woon, W-Y.. Nucleation and growth kinetics of multi-layered graphene on copper substrate. *Carbon* **135**, 118-124 (2018).
53. Zhang, J. et al. Preparation of bilayer graphene utilizing CuO as nucleation sites by CVD method. *J. Mater. Sci. Mater. Electron.* **29**, 4495-4502 (2018).
54. Qi, Z. et al. Chemical vapor deposition growth of bernal-stacked bilayer graphene by edge-selective etching with H_2O. *Chem. Mater.* **30**, 7852-7859 (2018).
55. Cho, J. H., Na, S. R., Park, S., Akinwande, D., Liechti, K. M., Cullinan, M. A. Controlling the number of layers in graphene using the growth pressure. *Nanotechnol.* **30**, 235602 (2019).
56. Shen, C. et al. Criteria for the growth of large-area adlayer-free monolayer graphene films by chemical vapor deposition. *J. Materiomics* **5**, 463-470 (2019).
57. Liu, B. et al. Layer-by-Layer AB-stacked bilayer graphene growth through an asymmetric oxygen gateway. *Chem. Mater.* **31**, 6105-6109 (2019).
58. Chu, C-M., Woon, W-Y. Growth of twisted bilayer graphene through two-stage chemical vapor deposition. *Nanotechnology* **31**, 435603 (2020).
59. Chen, Q. et al. High-quality bilayer graphene grown on softened copper foils by atmospheric pressure chemical vapor deposition. *Sci. China Mater.* **63**, 1973-1982 (2020).
